# Decoding the multicellular ecosystem of vena caval tumor thrombus in clear cell renal cell carcinoma by single-cell RNA sequencing

**DOI:** 10.1186/s13059-022-02651-9

**Published:** 2022-03-31

**Authors:** Yue Shi, Qi Zhang, Hai Bi, Min Lu, Yezhen Tan, Daojia Zou, Liyuan Ge, Zhigang Chen, Cheng Liu, Weimin Ci, Lulin Ma

**Affiliations:** 1 Key Laboratory of Genomic and Precision Medicine, Beijing Institute of Genomics, and China National Center for Bioinformation, Chinese Academy of Sciences, Beijing, 100101 China; 2 University of Chinese Academy of Sciences, Beijing, 100049 China; 3 Department of Urology, Peking University Third Hospital, Beijing, 100191 China; 4 Department of Pathology, School of Basic Medical Sciences, Peking University Third Hospital, Peking University Health Science Center, Beijing, 100191 China; 5 Institute for Stem cell and Regeneration, Chinese Academy of Sciences, Beijing, China

**Keywords:** Tumor thrombus, Clear cell renal cell carcinoma, Single-cell RNA sequencing, Tumor heterogeneity, Tumor microenvironment

## Abstract

**Background:**

Vascular invasion with tumor thrombus frequently occurs in advanced renal cell carcinoma (RCC). Thrombectomy is one of the most challenging surgeries with high rate of perioperative morbidity and mortality. However, the mechanisms driving tumor thrombus formation are poorly understood which is required for designing effective therapy for eliminating tumor thrombus.

**Results:**

We perform single-cell RNA sequencing analysis of 19 surgical tissue specimens from 8 clear cell renal cell carcinoma (ccRCC) patients with tumor thrombus. We observe tumor thrombus has increased tissue resident CD8^+^ T cells with a progenitor exhausted phenotype compared with the matched primary tumors. Remarkably, macrophages, malignant cells, endothelial cells and myofibroblasts from TTs exhibit enhanced remodeling of the extracellular matrix. The macrophages and malignant cells from primary tumors represent proinflammatory states, but also increase the expression of immunosuppressive markers compared to tumor thrombus. Finally, differential gene expression and interaction analyses reveal that tumor-stroma interplay reshapes the extracellular matrix in tumor thrombus associated with poor survival.

**Conclusions:**

Our comprehensive picture of the ecosystem of ccRCC with tumor thrombus provides deeper insights into the mechanisms of tumor thrombus formation, which may aid in the design of effective neoadjuvant therapy to promote downstaging of tumor thrombus and decrease the perioperative morbidity and mortality of thrombectomy.

**Supplementary Information:**

The online version contains supplementary material available at 10.1186/s13059-022-02651-9.

## Background

There were more than 431,280 new kidney cancer cases diagnosed in 2020 worldwide [1], of which the most common histological subtype is clear cell renal cell carcinoma (ccRCC), accounting for approximately 70–80% of all renal cell carcinoma (RCC) cases [2–5]. One unique clinical aspect of RCC is that it can invade through the renal vein into the inferior vena cava (IVC), and even grow up to the right cardiac chambers [6]. Venous tumor thrombus (TT) in the renal vein or inferior vena cava was reported in approximately 15% of RCC patients [7]. Reese et al. showed the tumor thrombus level does not necessarily affect disease-specific survival, but has a major impact on the complexity of surgery with a significant risk of hemorrhage, unstable hemodynamics and death [8–11]. Neoadjuvant systemic therapy may potentially decrease the TT burden and thus improve the safety and feasibility of tumor thrombectomy potentially improving the curative potential of surgical resection [12–14]. However, the underlying mechanism of TT formation is poorly understood, which prevents the design of effective neoadjuvant therapy for downstaging tumor thrombus and decreasing the perioperative morbidity and mortality of thrombectomy.

Bulk genomic studies, including ours, have revealed that most TTs with the propensity of rapid growth [15], harbored limited additional genomic alterations compared with matched primary tumors (PTs) [16–18]. The lack of fixation of new driver events in TTs may be due to their rapid extension and/or limited selective pressure in the intravascular space. Notably, we previously showed that the cases with TTs had distinct transcriptomic profiles compared to those without TTs by unsupervised clustering analysis of bulk RNA-seq data [16]. But a limited number of differentially expressed genes were identified between PTs and paired TTs, while multiple pathways related to the immune response and extracellular matrix and structure were significantly enriched in TTs [16]. Moreover, a higher proportion of macrophages in PTs of patients with TTs than in those of patients without TTs was observed by CIBERSORT analysis [16]. Thus, not only cancer cells but also tumor-infiltrating cells may participate in the process of tumor thrombus. And the cell type-specific gene expression patterns will promote the understanding of the intratumoral heterogeneity and the biology of tumor thrombus, and help discover more effective targets for tumor thrombus therapy.

In this study, we dissected the tumor ecosystem in 19 surgical tissue specimens from 8 ccRCC patients (discovery cohort) by single-cell RNA sequencing (scRNA-seq). Moreover, we included the bulk RNA-seq data of 60 tumor thrombus and paired primary tumor specimens from 30 ccRCC patients with TTs [16], the CheckMate 025 cohort [19, 20], the Cancer Genome Atlas Kidney Renal Clear Cell Carcinoma (TCGA KIRC) and the IMmotion 150 cohort [21] as validation cohorts. We aimed to investigate the multicellular heterogeneity of tumor cell communities of tumor thrombus to provide valuable biological and clinical insights into this disease.

## Results

### scRNA-seq profiling of the tumor ecosystem in tumor thrombus and matched primary tumors

**Fig. 1:**
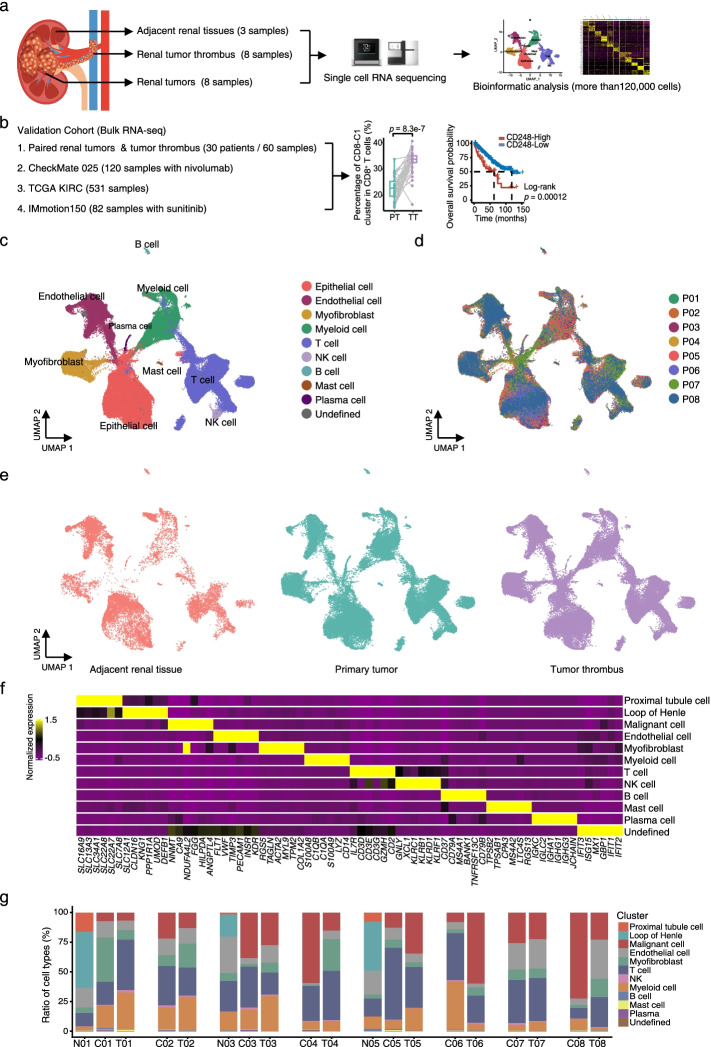
scRNA-seq profiling of the kidney primary tumors and tumor thrombus microenvironments. **a**, **b** Schematic diagram of the study strategy. The discovery cohort is shown in (**a**), and validation cohorts 1, 2, 3 and 4 are shown in (**b**). Validation cohort 1, 60 tumor thrombus and paired primary tumor specimens from 30 ccRCC patients. Validation cohort 2, 120 samples with advanced clear cell renal cell carcinoma with nivolumab treatment from CheckMate 025 cohort. Validation cohort 3, 531 samples of TCGA KIRC cohort. Validation cohorts 4, 82 samples with metastatic renal cell carcinoma with sunitinib treatment from IMmotion 150 cohort. **c** UMAP plot showing the annotation and color codes for cell types in the ccRCC ecosystem. Epithelial cell including normal epithelial cell (the proximal tubule cell and the loop of Henle cell) in ARTs and malignant epithelial cell in PTs and TTs. **d**, **e** The UMAP plot showing cell origins by color, patient origin (**d**), and ART, PT or TT origin (**e**). **f** Heatmap showing the expression of marker genes in the indicated cell types. **g** Histogram indicating the proportions of cells in tissues of each patient. N, adjacent renal tissues. C, primary tumors. T, tumor thrombus

To elucidate the tumor ecosystem in venous tumor thrombus, we collected 19 surgical tissue specimens (discovery cohort) from treatment-naïve ccRCC patients (ccRCC, *n* = 8) for scRNA-seq. Tumor thrombus (*n* = 8), paired primary tumors (*n* = 8) and adjacent renal tissues (*n* = 3) were referred to as TTs, PTs and ARTs, respectively (Fig. 1a and Additional file 2: Table S1). Moreover, we included 4 different cohorts with bulk RNA-seq data for further validation (Fig. 1b). Approximately 1 billion unique transcripts were obtained from 140,805 cells: 62,035 cells from TTs, 58,695 cells from PTs, and 20,075 cells from ARTs. After filtering, a total of 120,780 cells were used for further analysis (Additional file 1: Fig. S1a-c). We identified and visualized 9 major cell types according to the expression of canonical gene markers using the Uniform Manifold Approximation and Projection (UMAP) method [22] (Fig. 1c-f and Additional file 1: Fig. S1a-c): epithelial cells, T cells, natural killer (NK) cells, myeloid cells, myofibroblasts, endothelial cells (ECs), B cells, plasma cells and mast cells. For the 16 PT and TT samples, all these cell subtypes were shared among patients and between PTs and TTs with no consistent change, albeit at different proportions (Fig. 1g). For the ART samples, in addition to the immune and stromal cells, we obtained a total of 6809 normal epithelial cells and further identified as the proximal tubule cells and the loop of Henle cells based on the classic markers, as described previously [22] (Fig. 1f-g and Additional file 1: Fig. S1d). In summary, our results revealed substantial heterogeneity of ecosystem compositions among the ART, PT and TT samples.

### T/NK cell clustering and state analysis identifies tumor thrombus are enriched with tissue resident CD8^+^ T cells in a progenitor exhausted state compared to primary tumors

**Fig. 2:**
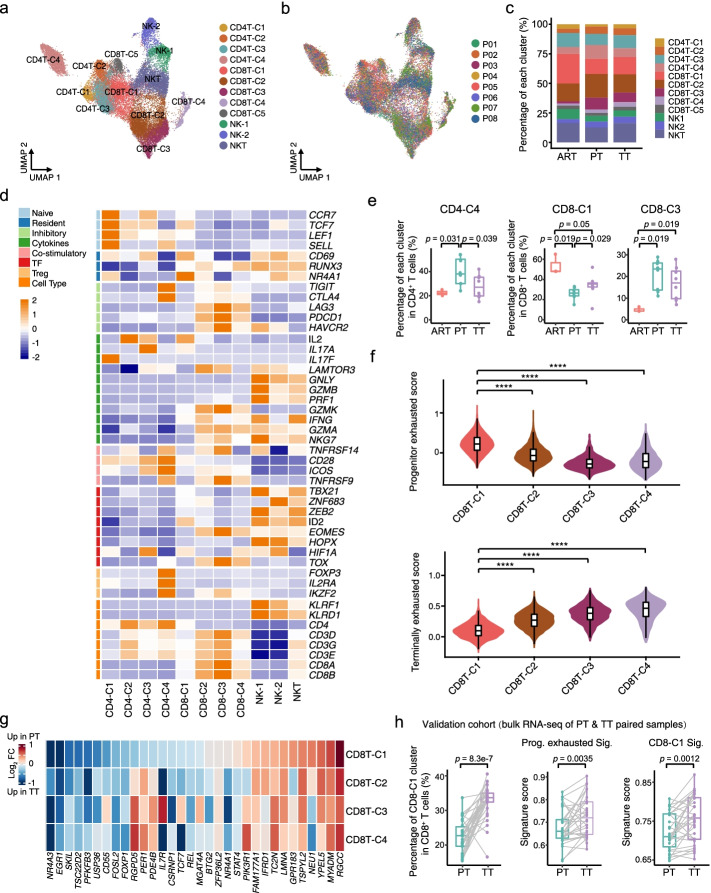
scRNA-seq revealed heterogeneity in T/NK cells in primary tumors and tumor thrombus. **a** UMAP plot showing the subtypes of T/NK cells derived from ART, PT and TT samples. Each cluster is color-coded according to cell type. **b** UMAP plot illustrating T/NK cells clustered and color-coded according to each patient. **c** Bar plot illustrating the fraction of T/NK subgroups in ARTs, PTs and TTs. **d** Heatmap indicating the expression of selected gene sets in T/NK subtypes, including naïve, resident, inhibitory, cytokine, costimulatory, transcription factor (TF), and cell type. **e** Box plots illustrating the average proportion of CD4-C4, CD8-C1, and CD8-C3 subtypes among ARTs, PTs and TTs. *p* values were determined by a two-sided Wilcoxon test. **f** Violin and box plots showing the signature score distribution for progenitor exhausted and terminally exhausted CD8^+^ T cells within CD8-C1 to CD8-C4 subsets. *p* values were determined by a two-sided Wilcoxon test. ****, *p* < 0.0001. **g** Heatmap showing the fold change in the expression of the progenitor exhausted gene set of CD8-C1 to CD8-C4 cells in PTs compared with TTs. **h** Box plots representing the percentage of CD8-C1 and the scores of progenitor exhausted signature and CD8-C1 signature in paired PT and TT bulk RNA-seq samples. The CD8-C1 cell fraction per sample as inferred by CIBERSORTx. *p* values were determined by a two-sided Wilcoxon test

Considering that recent studies have observed dramatic reduction of tumor thrombus in ccRCC patients after ICB therapy [23–26], we speculated that tumor-infiltrating immune cells may play important roles in the process. Therefore, we first performed unsupervised clustering of T and NK cells and obtained 12 clusters across ARTs, PTs and TTs, including four subtypes of CD4^+^ T cells (CD4-C1 to CD4-C4), five subclusters of CD8^+^ T cells (CD8-C1 to CD8-C5), two NK subclusters (NK1 and NK2) and one NKT cluster (Fig. 2a-d, Additional file 1: Fig. S2a-d and Additional file 3: Table S2).

For CD4^+^ T cells, we identified naïve (CD4-C1; *CCR7*^+^), helper (CD4-C2; *IL2*^+^, CD4-C3; *IL7*^+^), and suppressive regulatory Treg (CD4-C4; *FOXP3*^+^) CD4^+^ T cells (Fig. 2d and Additional file 1: Fig. S2d). To determine the differences among ARTs, PTs and TTs, we calculated the percentage of each cluster in ARTs, PTs and TTs, and found that the relative percentage of naïve CD4^+^ T cells (CD4-C1) in PTs was reduced compared with those in ARTs and TTs (Additional file 1: Fig. S2e), and the proportion of suppressive Tregs (CD4-C4) was higher (Fig. 2e), indicating there might be in a more immune suppressive state in PTs.

For CD8^+^ T cells, we identified tissue resident (CD8-C1; *CD69*^+^), terminal exhausted (CD8-C2 and CD8-C3; *PDCD1*^+^ and *TOX*^+^), cycling (CD8-C4; *MKI67*^+^) and mucosal-associated invariant T (MAIT) (CD8-C5; *IL7R*^+^ and *CCR4*^−^) CD8^+^ T cells (Fig. 2d and Additional file 1: Fig. S2c-d) according to the expression of canonical markers [27–31]. Similar with the proportion of CD4-C1 cells (Additional file 1: Fig. S2e), the abundance of CD8-C1 cells was also increased in TTs compared with PTs, but much less in both PTs and TTs than in ARTs (Fig. 2e). Although a considerable amount of tissue resident CD8^+^ T cells has been found in normal kidney tissues [32–34], the mechanisms for it are still largely unknown. Consistent with our finding, another study about hepatocellular carcinoma (HCC) also revealed the tissue resident CD8^+^ T cells were more abundant in adjacent non-tumor tissues than in tumors [35]. Furthermore, as observed previously in ccRCC [36], the proportion of exhausted CD8^+^ T cells (CD8-C3) was increased in tumor tissues, but with no significant difference between PTs and TTs (Fig. 2e and Additional file 1: Fig. S2e), indicating that CD8^+^ T cells are exhausted in both PTs and TTs. In pseudotime analysis, we removed MAIT cells, due to their differing TCR characteristics [35]. The continuous developmental trajectory of CD8-C1 to C4 represented a binary branched structure: CD8-C1 was the root, CD8-C3 and C4 were the end states of the two branches, and CD8-C2 was in a transitioning state (Additional file 1: Fig. S2f), which were very similar between PTs and TTs. Pathway analysis from the trajectory indicated T cell activation and lymphocyte differentiation was upregulated at an earlier stage, and cell cycle, ATP metabolism and hypoxia related pathways were highly enriched in the end state of CD8^+^ T cells (Additional file 1: Fig. S2g). These results suggested the CD8^+^ T cells shared the same transition trajectory between PTs and TTs, along with dysfunctional and metabolic disorders, and finally turned to be exhausted in both PTs and TTs.

There are evidences showed that certain CD8^+^ T cells in the progenitor exhausted state could enhance the efficacy of immune checkpoint blockade (ICB) therapy in melanoma and kidney cancer [37, 38]. Meanwhile, considering a striking regression of TTs with ICB therapy in some ccRCC patients [23–26], we asked whether there was certain subset of CD8^+^ T cells we recovered may resemble the progenitor exhausted phenotype. Then, we scored the CD8^+^ T cell subclusters for progenitor and terminally exhausted gene signatures according to the published studies [37, 38]. We noticed that the progenitor exhausted signature was significantly enriched in CD8-C1 subcluster, and the terminally exhausted signature was obviously lower than that of other subsets (Fig. 2f). Moreover, most of the progenitor exhausted signature genes showed upregulated expression in CD8-C1 cells in TT samples (Fig. 2g), which further demonstrated the progenitor exhausted phenotype was enriched in TTs. Furthermore, we examined a larger cohort of patients with paired PT and TT bulk transcriptomes and observed significantly increased proportions of the CD8-C1 subset in TTs compared with PTs, and the progenitor exhausted and CD8-C1 signature scores were both upregulated in TTs (Fig. 2h). Having identified CD8-C1 cells (the tissue resident CD8^+^ T cells) and its signature were enriched more in TTs than in PTs, we next examined whether CD8-C1 cells we found will show a better response to anti-PD-1 therapy by using the pretherapy bulk RNA-seq data from a larger cohort of ccRCC patients (CheckMate 025) treated with nivolumab (anti-PD-1) [19, 20]. As expected, the high levels of the CD8-C1 signature were associated with improved progression free survival (PFS) (Additional file 1: Fig. S2h), which was also supported by another study published recently that they observed a strong enrichment of the tissue resident CD8^+^ T cell cluster in one ccRCC patient with complete response to ICB [31]. Collectively, these results suggested that a high signature of tissue resident CD8^+^ T cells in both primary ccRCC and tumor thrombus may be the hint of a good response to ICB therapy.

### Macrophages show enhanced ECM remodeling activity in tumor thrombus, and immune-reactive perturbations in primary tumors

**Fig. 3:**
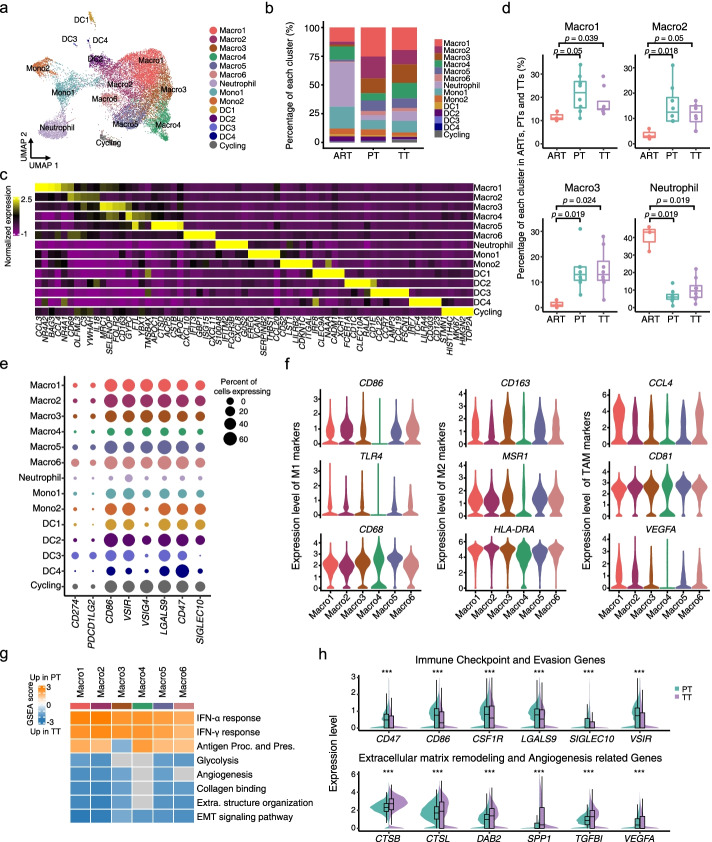
Detailed characterization of myeloid cells in primary tumors and tumor thrombus. **a** UMAP plot showing the subtypes of myeloid-derived cells derived from ART, PT and TT samples. Each cluster is color-coded according to cell type. **b** Bar plot illustrating the average proportion of each myeloid subtype among ARTs, PTs and TTs. **c** Heatmap showing the expression of marker genes in each subtype of myeloid-derived cells. **d** Box plots illustrating the fraction of the subclusters of Macro1–3 and neutrophils in ARTs, PTs and TTs. *p* values were determined by a two-sided Wilcoxon test. **e** Dot plot showing the percentage of cells and expression level in each cell type expressing immune checkpoint and evasion-related genes. **f** Violin plots representing the expression levels of M1, M2 and TAM marker genes in macrophage subtypes. *p* values were determined by a two-sided Wilcoxon test. **g** Heatmap of GSEA scores indicating the pathways significantly enriched in PTs or TTs. INF-γ, interferon- γ. INF-α, interferon- α. Antigen Proc. and Pres., antigen presentation and processing. Extra. structure organization, extracellular structure organization. EMT, epithelial-mesenchymal transition. **h** Violin and box plots comparing the expression distributions of immune checkpoint and evasion-related genes and ECM remodeling and angiogenesis-related genes between PTs and TTs. *p* values were determined by a two-sided Wilcoxon test. ***, *p* < 0.001

In addition to adaptive immunity, the innate immune cells might be a first barrier for rapid extension of TTs in the intravascular space, which may have important impacts on tumor thrombus formation and resistance to ICB therapy. Thus, we analyzed the transcriptomes of 16,267 myeloid cells in PTs (*n* = 7689), TTs (*n* = 7129) and ARTs (*n* = 1449). Myeloid cells exhibited remarkable heterogeneity and were categorized into 14 clusters. Based on the expression of canonical markers, we annotated these clusters into six subtypes for macrophages (Macro1-Macro6), two for monocytes (Mono1-Mono2), four for DCs (DC1-DC4), one for neutrophils and one for cycling cells (Fig. 3a-c, Additional file 1: Fig. S3a-d and Additional file 4: Table S3). Macrophages were enriched in PTs and TTs compared with ARTs, except Macro4 cluster (Fig. 3b, d and Additional file 1: Fig. S3e). Neutrophils were remarkably depleted in both PTs and TTs compared with ARTs (Fig. 3d), implying tumor cells of PTs and TTs may escape elimination by circulating neutrophils. To better understand the roles of these myeloid-related populations, we first examined the immune checkpoint- and evasion-related gene expression levels. Almost all these genes were highly expressed in the myeloid subpopulations except neutrophils (Fig. 3e). While *CD274* (*PD*-*L1*) and *PDCD1LG2* (*PD*-*L2*), both ligands for PD-1 signaling mediating immune checkpoint in T cells, were detected sparsely across all myeloid subpopulations (Fig. 3e). These results suggested that neutrophils might be more effective in confronting cancer cells, and other macrophages, monocytes and DCs might already be ‘educated’ by PT or TT tumor cells.

Macrophages are usually classified into two canonical subtypes, proinflammatory M1 and anti-inflammatory M2 [39–41]. However, we could not clearly distinguish M1 and M2 macrophages by known marker genes such as *CD86*, *TLR4* (M1) and *CD163* and *MSR1* (M2), as they were all expressed in Macro1-Macro3 and Macro5-Macro6 but poorly expressed in Macro4 (Fig. 3f). While the tumor-associated macrophage (TAM) markers, as well as *CD68* and *HLA*-*DRA* as both M1/M2 and tumor-associated macrophage (TAM) markers were highly expressed in each macrophage subtype (Fig. 3f), indicating they were all TAMs. Intriguingly, we noticed a significant enrichment of gene expression signatures in the proinflammatory phenotype, such as response to interferon, and antigen presentation pathways in TAMs of PTs compared to that of TTs (Fig. 3g and Additional file 1: Fig. S3f). Surprisingly, these TAMs in PTs also upregulated expression of immune checkpoint and evasion markers compared to TTs (Fig. 3h). Thus, TAMs in PTs exhibited both proinflammatory and immune suppressive phenotypes, potentially contributing to tumor cells immune escape. Whereas, in TTs, we observed increased signature scores associated with tumor progression-related pathways, including epithelial-mesenchymal transition (EMT), extracellular structure organization and angiogenesis (Fig. 3g-h and Additional file 1: Fig. S3f). These findings were consistent with previous reports that macrophages act as shapers of the tumor ECM to facilitate cell migration and angiogenesis [42, 43]. Collectively, the complexity of TAMs demonstrated distinct dysfunctional states between PTs and TTs, with a more immunosuppressive phenotype in PTs, and enhanced ECM remodeling in TTs.

### Malignant cells exhibited increased ECM remodeling in tumor thrombus, and a more immunosuppressive phenotype in primary tumors

**Fig. 4:**
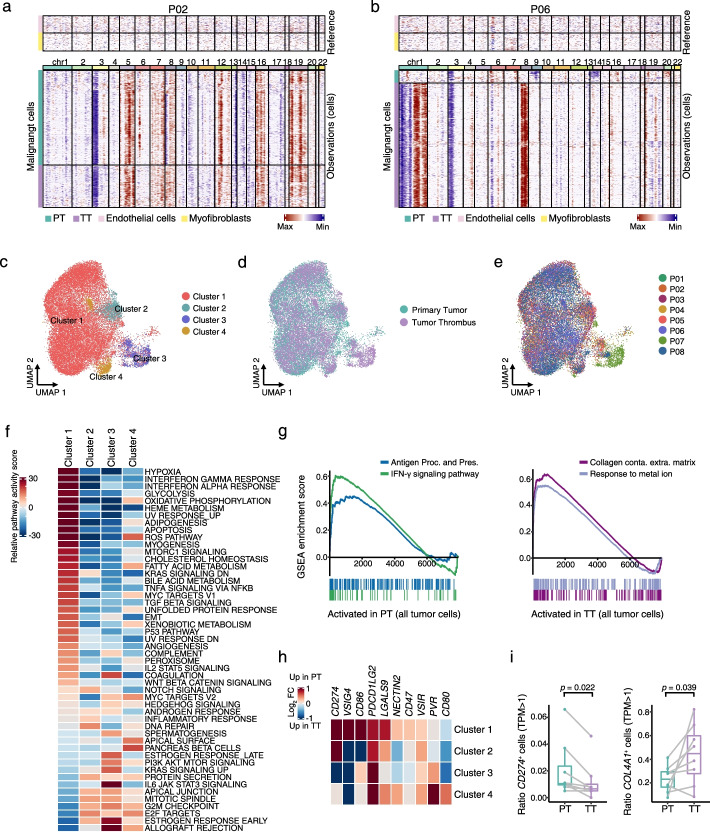
Identification and characterization of malignant cells in primary tumors and tumor thrombus. **a**, **b** Heatmaps showing large-scale CNVs for individual malignant cells (rows) from P02 (left) (**a**) and P06 (right) (**b**). Endothelial cells and myofibroblasts were treated as references (top), and malignant cells were observed in PTs and TTs (bottom). The color bar red indicates amplifications, and blue indicates deletions. **c** UMAP plot representing the subtypes of malignant cells from PT and TT samples. **d** UMAP plot showing malignant cells derived from PTs and TTs, colored by tissue origin. **e** UMAP plot illustrating color-coded tumor cells, according to each patient. **f** Differentially expressed pathways were scored per cell by GSVA among 4 malignant cell subtypes. **g** GSEA of the interferon-γ (IFN-γ) signaling pathway and antigen presentation and processing (Antigen Proc. and Pres.) enrichment scores in all malignant cells in PTs compared with in TTs; collagen containing extracellular matrix (Collagen conta. Extra. matrix) and response to metal ion enrichment scores in all tumor cells in TTs compared with in PTs. **h** Heatmap depicting the differential expression fold change of PTs compared with TTs for immune checkpoint and evasion-related genes. **i** Box plots showing the fraction of *CD274*^+^ and *COL4A1*^+^ tumor cells in PT and TT single-cell samples. *p* values were determined by a two-sided Wilcoxon test

To better understand cellular programs active in cancer cells that may function together with the immune cells, we next sought to identify the expression patterns of the malignant cells between PTs and TTs. The tumor cells were confirmed by the detection of copy number variations (CNVs), inferred by scRNA-seq data, using myofibroblasts and endothelial cells as references, according to a published study [44]. Seven of 8 patients showed similar CNV patterns and subclonal structures between TTs and PTs, except patient P06 (Fig. 4a-b and Additional file 1: Fig. S4a), supporting the previous studies of limited additional genomic alterations in TTs [16, 18]. Then, we evaluated the transcriptional difference of malignant cells between PTs and TTs. In total, we obtained 15,012 malignant epithelial cells from PTs and 12,078 cells from TT specimens. Since the proximal tubule cells has been identified as the origin of ccRCC [22], we then compared the hypoxia and angiogenesis signatures, which were two of classic biological states contributed to carcinogenesis, across the malignant cells and the proximal tubule cells. Consistent with previous studies, both PT and TT malignant cells had significantly higher hypoxia and angiogenesis signatures than cells from ARTs (Additional file 1: Fig. S4b). Then, focusing on the malignant cells, we totally obtained 4 clusters, and the proportion in individual patients was different in each subcluster, further supporting a high degree of intertumoral heterogeneity (Fig. 4c-e and Additional file 1: Fig. S4c-f). To better understand the transcriptional heterogeneity of them, we scored the four clusters by GSVA Hallmark analysis, and observed dramatic transcriptional program differences among each cluster (Fig. 4f). In detail, cluster 1 was the most abundant and characterized with increased hypoxia, glycolysis, oxidative phosphorylation (OXPHOS), along with concomitant immune activation and metabolic pathways (Fig. 4f). The coexistence of glycolysis and OXPHOS metabolic states in ccRCC has been reported in another study [38] in which the hybrid phenotype in RCC contributes to metabolic plasticity, allowing cancer cells to adapt to various microenvironment [45–47]. Furthermore, interestingly, similar with the TAMs, we observed an enrichment of genes involved in interferon-γ (IFN-γ) response and antigen presenting pathways with upregulated expression of immune checkpoint and evasion genes in PTs compared to TTs (Fig. 4g-h, Additional file 1: Fig. S4g-h and Additional file 5: Table S4). It demonstrated their capacity to promote immune escape and repress the anti-tumor immune response. While, the TT tumor cells were activated in ECM remodeling and response to metal ion related pathways (Fig. 4g and Additional file 1: Fig. S4g-h). Consistent with these results, a significant higher proportion of *CD274*^+^ malignant cells in PTs, and more *COL4A1*^+^ tumor cells in TTs were observed (Fig. 4i), indicating the higher immunosuppressive state in PTs and the dynamic remodeling of ECM in TTs. Furthermore, we found the classic ferroptosis suppressor gene *GPX4* showed upregulated expression in TTs (Additional file 1: Fig. S4h), implying inducing ferroptosis through regulation of *GPX4* for tumor therapy might be more effective in TTs than in PTs in ccRCC patients. In summary, at single-cell resolution, we revealed the malignant cells in PTs demonstrated a stronger immunosuppressive phenotype, while tumor cells in TTs showed a higher ECM reprogramming state.

### Endothelial cells represent upregulation of ECM remodeling related pathways in tumor thrombus, and highly-abundant *CCL4*^*+*^ and *NDUFA4L2*^+^ endothelial cells are associated with poor survival

**Fig. 5:**
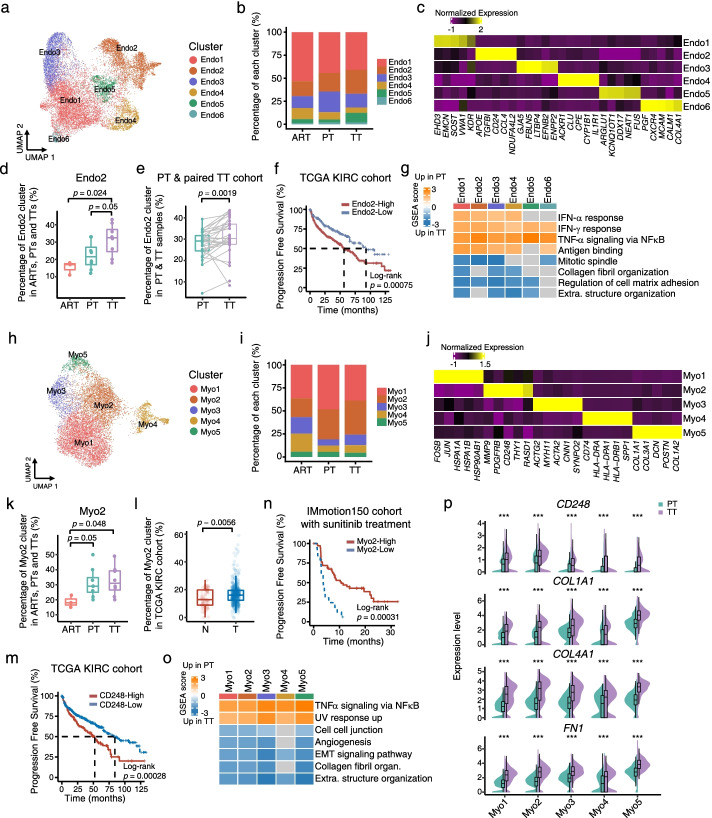
Detailed characterizations of endothelial cells and myofibroblasts in ARTs, PTs and TTs. **a** UMAP plot showing the subtypes of endothelial cells derived from ART, PT and TT samples. Each cluster is color-coded according to cell type. **b** Bar plot illustrating the average proportion of each endothelial subtype among ARTs, PTs and TTs. **c** Heatmap showing the expression of marker genes in each subtype of endothelial cells. **d** Box plots illustrating the fraction of Endo2 subgroup in ARTs, PTs and TTs. *p* values were determined by a two-sided Wilcoxon test. **e** Box plots showing the percentage of Endo2 in paired PT and TT bulk RNA-seq samples, inferred by CIBERSORTx. *p* values were determined by a two-sided Wilcoxon test. **f** Kaplan-Meier plot showing that KIRC patients in the TCGA dataset with high expression of Endo2 signature had shorter progression free survival (PFS). **g** Heatmap of GSEA scores indicating the pathways significantly enriched in PTs or TTs. Extra. structure organization, extracellular structure organization. **h** UMAP plot showing the subtypes of myofibroblasts derived from the ART, PT and TT samples. Each cluster is color-coded according to cell type. **i** Bar plot illustrating the average proportion of each myofibroblast subtype among ARTs, PTs and TTs. **j** Heatmap showing the expression of marker genes in each subtype of myofibroblasts. **k** Box plot illustrating the average proportion of Myo2 subcluster among ARTs, PTs and TTs. *p* values were determined by a two-sided Wilcoxon test. **l** Box plot representing the proportions of subclusters of Myo2 in myofibroblasts between normal (N) and tumor (T) samples in the TCGA cohort. *p* values were determined by a two-sided Wilcoxon test. **m** Kaplan-Meier plot showing that KIRC patients in the TCGA dataset with high expression of *CD248* had worse progression free survival (PFS). **n** Kaplan-Meier plot showing that patients from the sunitinib arm of the IMmotion150 cohort with a high Myo2 signature score had improved progression free survival. **o** Heatmap of GSEA enrichment scores for indicating gene sets significantly enriched in PTs or TTs. Collagen fibril organ, collagen fibril organization. Extra. structure organization, extracellular structure organization. EMT, epithelial- mesenchymal transition. **p** Violin and box plots demonstrating the expression levels of *CD248*, *COL1A1*, *COL4A1*, and *FN1* between PTs and TTs. *p* values were determined by a two-sided Wilcoxon test. ***, *p* < 0.001

To further evaluate endothelial cells roles that may facilitate tumor thrombus growth and extension in the intravascular space, we focused on endothelial cells (ECs), and obtained 16,221 ECs from PTs (*n* = 4808), TTs (*n* = 7915) and ARTs (*n* = 3498). These cells were subclustered into six different clusters (Fig. 5a-c and Additional file 1: Fig. S5a-c), and each of them was identified based on the classically expressed genes in ECs [48, 49], including glomerular like ECs (Endo1: *SOST*^*+*^), cancer-related ECs (Endo2: *NDUFA4L2*^+^), arterial ECs (Endo3: *GJA5*^*+*^), *ACKR1*^*+*^ ECs (Endo4: *ACKR1*^*+*^), the tip cells (Endo6: *CXCR4*^*+*^), and one undefined cluster (Endo5) (Fig. 5a-c, Additional file 1: Fig. S5a-c and Additional file 6: Table S5). Furthermore, we found the ratio of Endo2 in TTs was higher than that in ARTs and PTs (Fig. 5d and Additional file 1: Fig. S5d), suggesting that cancer related vasculature formation may facilitate the rapid extension of TTs in the intravascular space. In support, we observed significantly increased percentages of Endo2 subset in TTs through analyzing the bulk transcriptomes from a large cohort of patients with 30 paired PTs and TTs (Fig. 5e). Moreover, the high levels of Endo2 gene signature were significantly associated with poor survival in TCGA KIRC cohort (Fig. 5f and Additional file 1: Fig. S5e). Interestingly, like TAMs and malignant cells, we found significantly increased expression signatures associated with interferon response and antigen binding pathways in ECs of PTs (Fig. 5g and Additional file 1: Fig. S5f-g), but along with variation in genes expression related to immunosuppression among the subsets of ECs between PTs and TTs (Additional file 1: Fig. S5h). At the same time, we noticed in Endo2 subset, the expression level of most of genes related to immunosuppression was upregulated in PTs compared with TTs (Additional file 1: Fig. S5h), suggesting these cells in PTs might be in a more immunosuppressive state. Furthermore, we found that the ECs had enhanced ECM remodeling and cell proliferation activities in TTs (Fig. 5g and Additional file 1: Fig. S5f-g). Thus, these data indicated that in addition to TAMs and cancer cells in TTs, endothelial cells in TTs may modify the ECM to facilitate tumor thrombus growth, and the molecular therapies targeting the cells with Endo2 signature might be able to specifically downstage the vena cava thrombus.

### Myofibroblasts in tumor thrombus enhance ECM remodeling with increased extracellular matrix production compared with primary tumors

Fibroblasts have been shown to be a major contributor to ECM remodeling in tumor progression in previously studies. Thus, we further investigated fibroblasts in ARTs, PTs and TTs. Almost all fibroblasts were positive for α-SMA, a conventional marker of myofibroblasts (Fig. 1f, Additional file 1: Fig. S1c), which is consistent with the findings in a previous ccRCC report [36]. We found five distinct subtypes by reclustering 10,726 myofibroblasts, according to the published studies [36, 50] (Fig. 5h-j, Additional file 1: Fig. S5i-k and Additional file 6: Table S5). In detail, our findings identified a state of stress with highly expressed heat shock proteins in Myo1, a highly vascular pericyte-like phenotype in Myo2, *ACTG2*^+^ myofibroblasts association in Myo3, antigen presentation phenotype in Myo4, and the characteristics of cancer-associated myofibroblasts in Myo5 [50]. Moreover, we found that vascular pericyte-like Myo2 was enriched in TTs and PTs compared to ARTs (Fig. 5k and Additional file 1: Fig. S5l), suggesting that Myo2 may help to remodel tumor vessels toward a mature phenotype in tumor thrombus, since pericytes are necessary for vessel maturation [51, 52]. Furthermore, we validated this result in the TCGA KIRC cohort. Indeed, the percentage of Myo2 increased significantly in the tumor tissues of KIRC patients compared to normal tissues (Fig. 5l). In addition, the marker gene of Myo2, *CD248,* was remarkably upregulated in tumors compared to the adjacent tissues (Additional file 1: Fig. S5m), and significantly associated with poor survival in KIRC patients (Fig. 5m and Additional file 1: Fig. S5n). Antiangiogenic tyrosine kinase inhibitors (TKIs) have been broadly applied in ccRCC patients, and pericytes play crucial roles in integrity of the tumor microvasculature [52–55]. Therefore, we further evaluated whether the proportion of Myo2 could predict the response to TKIs. We found in the IMmotion150 cohort [21], patients with sunitinib treatment had an improved PFS with high score of Myo2 signature (Fig. 5n), indicating that Myo2 might be a good predictor of response to TKIs in ccRCC patients.

Next, we examined whether there were also transcriptional differences in myofibroblasts between TTs and PTs, and found all the myofibroblast subclusters in PTs were enriched in the TNFα and UV response pathways, while almost all myofibroblasts in TTs increased the signatures associated with angiogenesis and ECM remodeling, including extracellular matrix organization, collagen fibril organization and EMT pathways (Fig. 5o-p and Additional file 1: Fig. S5o). Therefore, myofibroblasts, like TAMs, malignant cells and ECs, may also play promoting roles in ECM remodeling of TTs, thereby possibly representing a potential target for ccRCC patients with TTs.

### Tumor-stroma interplay reshaped the extracellular matrix in tumor thrombus associated with poor survival

**Fig. 6:**
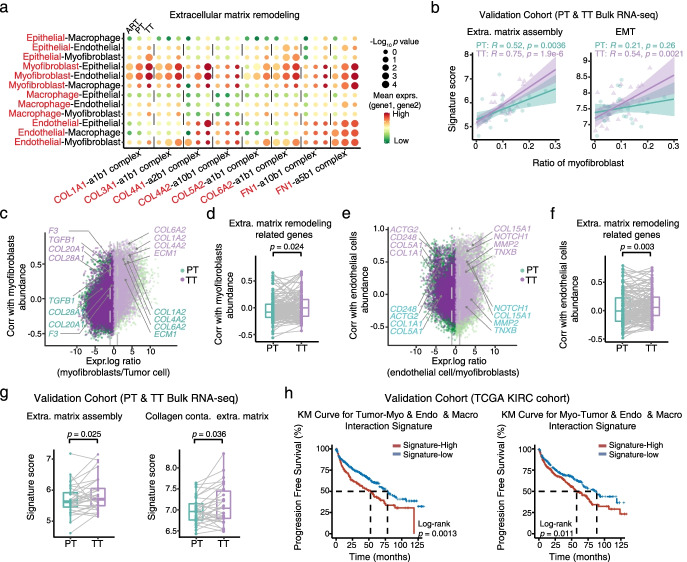
Tumor-stroma interplay reshaped the extracellular matrix in tumor thrombus associated with poor survival. **a** Dot plot showing inferred interactions between epithelial cells (proximal tubule cells from ARTs and malignant cells from PTs and TTs) and macrophages, endothelial cells, and myofibroblasts. Circle size indicates the significance of the interaction, and circle color indicates the mean expression of ligand and receptor genes. The red letters represent ligands, and the black letters represent receptors. **b** Scatterplots demonstrating the extracellular matrix assembly (left) and EMT (right) signature score versus total myofibroblast cell fraction in bulk RNA-seq of PT-TT paired samples in the validation cohort. Cell-type fractions were inferred using CIBERSORTx. The Pearson coefficient (R) and associated *p* value are reported for each correlation. **c** Scatterplot depicting inferred cell-to-cell interactions between tumor cells and myofibroblasts by combining scRNA-seq data and bulk RNA-seq data. **d** Box plot showing the calculated correlation between extracellular matrix remodeling-related genes and myofibroblast relative abundance involved in PTs and TTs. *p* values were determined by a two-sided Wilcoxon test. **e** Scatterplot representing inferred cell-to-cell interactions between myofibroblasts and endothelial cells by combining scRNA-seq data and bulk RNA-seq data. **f** Box plot showing the calculated correlation between extracellular matrix remodeling-related genes and endothelial cell relative abundance involved in PTs and TTs. *p* values were determined by a two-sided Wilcoxon test. **g** Signature scores for extracellular matrix assembly (left) and collagen-containing extracellular matrix (right) in paired PT-TT bulk RNA-seq samples in the validation cohort. *p* values were determined by a two-sided Wilcoxon test. **h** Progression free survival for the TCGA KIRC cohort based on high tumor-stroma and stroma-tumor interaction signatures versus low signature expression

Given the observations of activated ECM remodeling as common trends in macrophages, malignant cells, ECs and myofibroblasts of TTs compared to PTs, we hypothesized that the different cell populations participated in complex crosstalk. To identify possible non-cell-autonomous effects, we used CellPhoneDB to identify putative signaling between different cell populations via known receptor-ligand pairs (Additional file 7: Table S6). Notably, the interactions in multiple ligand-receptor pairs within the ECM remodeling pathway were stronger in TTs than in ARTs and PTs, and myofibroblasts were predicted to have the strongest signals of significant interactions with tumor cells and ECs, regardless of subgroups of them (Fig. 6a and Additional file 1: Fig. S6a). We additionally validated this finding in the validation cohort with 30 paired PT and TT bulk RNA-seq data. Consistent with the upregulated expression of ECM components and their receptors in stromal cells and other types of cells in TTs, the ECM assembly signature was robustly more strongly correlated with the CIBERSORTx-estimated myofibroblast fraction in TTs compared with in PTs (Fig. 6b and Additional file 1: Fig. S6b), supporting that the ECM remodeling related pathways were more activated in TTs than that in PTs.

Given the strong interactions identified between myofibroblasts and malignant cells and endothelial cells, we first examined whether tumor cells governed stromal myofibroblasts recruitment. We combined the single-cell transcriptomes with bulk RNA-seq according to previous studies [56, 57]. For example, we searched for genes increased in tumor cells rather than in myofibroblasts in single-cell data that were correlated with myofibroblast abundance. In detail, we found extensive interactions between myofibroblasts and tumor cells. In detail, tumor cells upregulated *COL20A1*, *COL28A1* and *TGFB1* expression to recruit more myofibroblasts in TTs compared with in PTs (Fig. 6c). Furthermore, myofibroblasts released collagen-related genes such as *COL6A2*, *COL1A2* and *COL4A2* to promote extracellular matrix assembly and have higher correlation with the abundance of myofibroblasts in TTs (Fig. 6c). The correlation between the relative abundance of myofibroblasts and extracellular matrix remodeling-related genes up-regulated in tumor cells was significantly higher in TTs than in PTs (Fig. 6d and Additional file 1: Fig. S6c). These data suggest that tumor cells might recruit more myofibroblasts in TTs by secreting ECM-related components. Additionally, we also found that myofibroblasts conferred enhanced recruitment of ECs in TTs than that in PTs, which might potentially facilitate angiogenesis (Fig. 6e-f and Additional file 1: Fig. S6d). Furthermore, the expression signature scores of ECM assembly, collagen-containing ECM and extracellular structure organization were all at a higher level in TTs than that in PTs in the validation cohort of 30 paired PT and TT ccRCC patients (Fig. 6g and Additional file 1: Fig. S6e). Thus, we showed that the stronger tumor-stroma interplay in the tumor thrombus was accompanied by the upregulated expression of a specific set of ECM related genes and pathways in both tumor cells and stromal cells.

To further investigate the association between tumor-stroma interplay and patient prognosis, we defined two interaction signature scores using significant L-R pairs to calculate the strengths of the ECM remodeling-related interactions in the TCGA KIRC cohort. The tumor-stroma interaction score was calculated between tumor cells and myofibroblasts, endothelial cells, and macrophages, while, the stroma-tumor interaction score was generated between myofibroblasts and tumor cells, endothelial cells, and macrophages. Notably, we found a stronger tumor-stroma interplay was associated with poor survival (Fig. 6h and Additional file 1: Fig. S6f). In summary, tumor-stroma interplay reshaped the extracellular matrix in tumor thrombus associated with poor survival.

## Discussion

Surgical treatment of locally advanced ccRCC with TTs, especially with those that extend to the inferior vena cava, remains a clinical challenge. Neoadjuvant targeted therapies, such as VEGFR or mTOR inhibitors, have been shown to reduce tumor thrombus levels and potentially make thrombectomy more feasible. However, the percentage of benefited patients showing thrombus regression from these treatments were extremely variable (from 25 to 67%) in different clinical studies [12]. Specifically, neoadjuvant immunotherapy was only effectively reducing the tumor thrombus size in some patients [23–26]. Thus, systematically deciphering the molecular differences between tumor thrombus and primary tumors is urgently needed for the development of new predictive markers and more effective therapies for ccRCC with TTs. Here, we presented a comprehensive single-cell transcriptomic atlas characterizing the heterogeneity of tumor cells, immune cells, and stromal cells in primary tumors and paired tumor thrombus. Cell type-specific biological features associated with the rapid extension of tumor thrombus in the intravascular space were also identified, which are potentially useful in designing more effective therapies for patients with tumor thrombus.

Our results showed that macrophages, malignant cells, endothelial cells and myofibroblasts in TTs exhibited enhanced remodeling of the extracellular matrix pathways compared to matched primary cancer cells, and the tumor-stroma interplay reshaped the extracellular matrix in tumor thrombus and was associated with poor survival. These results suggest that malignant cells of TTs are not independently suspended in the blood vessels, but require to form an ECM network embedded with immune cells and stroma cells to promote tumor thrombus growth. Although the cancer cells of TTs have intravasated into vein, and might have more opportunity to spread to lung, there is no direct experimental or clinical evidence that ccRCC patients with tumor thrombi are more likely to develop pulmonary metastasis. Previous studies demonstrated the ECM is important at initial stages of metastasis, where interactions between tumor cells and extracellular matrix induce an invasive phenotype [58, 59]. Moreover, TT grade has been reported as an independent predictor for metastatic outcome irrespective of the grade of the PTs [60], which suggests the invasion ability of tumor cells in TTs is very important for metastatic potential. Our study demonstrated that ECM remodeling is crucial for both metastasis and tumor thrombus growth. Thus, targeting ECM remodeling factors associating with TT formation might represent effective counter-measurements in ccRCC patients with TTs.

The high efficacy of ICB therapies in some ccRCC patients with tumor thrombus suggest the need to identify what kind of features or markers are likely to benefit from ICBs, based on a better understanding of the immune microenvironment between the primary tumor and tumor thrombus. Our data revealed that TAMs did not polarize to either M1-like or M2-like subsets, but showed distinct molecular and metabolic phenotypes with no subset diversity between PTs and TTs. Interestingly, TAMs from PTs showed both pro-inflammatory and anti-inflammatory phenotypes compared with TTs, suggesting TAMs in PTs may potentially facilitate the escape of tumor cells from immune surveillance. Importantly, more abundant subcluster of CD8^+^ T cells with the progenitor exhausted state in TTs was further validation in a bulk RNA-seq cohort. These cells have similarities to those described as mediating ICB response in melanoma and advanced RCC patients [38, 61]. Our findings may provide a mechanistic explanation as to why some patients with TTs respond to anti-PD-1 immunotherapy effectively. Moreover, we detected an association between pre-therapy levels of tissue resident CD8^+^ T cell signature and better response to anti-PD-1 therapy, suggesting that anti-PD-1 immunotherapy may obtain a greater benefit in reducing both primary tumor and tumor thrombus with higher proportions of tissue resident CD8^+^ T cells. Furthermore, these findings also suggest a potential value of tissue resident CD8^+^ T cell signature as a significant indicator for ICB response in ccRCC patients. Thus, strategies enhancing tissue resident CD8^+^ T cells may have good application prospects in immunotherapy for ccRCC patients with TTs. Moreover, it is necessary for further clinical and mechanistic investigation regarding the associations between tissue resident CD8^+^ T cells with ICB response in ccRCC patients with TTs given the small sample size of our single-cell and validation cohorts.

## Conclusions

In summary, this study provides evidence of phenotypic heterogeneity between primary tumors and tumor thrombus, in terms of tumor cells, immune cells, and stromal cells. Our data can be a valuable resource, facilitating a deeper understanding of the mechanisms associated with tumor thrombus and assisting in developing more effective neoadjuvant molecular therapies and biomarkers for advanced ccRCC patients with TT.

## Methods

### Clinical information and sample collection

All samples were obtained from Peking University Third Hospital, Beijing, China. Patient characteristics and clinical information are shown in Additional file 2 Table S1.

### Single-cell suspension preparation and sequencing

Single-cell suspensions for single-cell RNA-seq were obtained by mechanical and enzymatic dissociation. According to the manufacturer’s protocol, the Single Cell 3′ Library and Gel Bead Kit V3.1 (10X Genomics, 1,000,075) and Chromium Single Cell B Chip Kit (10x Genomics, 1,000,074) were used to prepare barcoded scRNA-seq. The libraries were finally sequenced using an Illumina NovaSeq6000 sequencer with a sequencing depth of at least 100,000 reads per cell with a paired-end 150 bp (PE150) reading strategy (performed by Capital Bio Technology, Beijing).

### scRNA-seq data processing

CellRanger (version 4.0.0) coupled with human reference version GRCh38 was used to process the 10X single-cell RNA-seq raw data for each sample, following the DoubletFinder R package (version 1.2.2) [62] to computationally infer and remove doublets in each sample individually, with default parameters. After removal of doublets, we employed the Seurat R package (version 3.2.2) [63] to analyze the output-filtered gene expression matrices. In brief, low-quality cells were removed if they met the following conditions: (i) > 5000 or < 200 genes and (ii) > 50% UMIs derived from the mitochondrial genome according published papers [22, 48, 64–67]. Then, according to the published studies [50, 65, 68–70], the filtered expression matrix was then normalized with the function “NormalizeData”, followed by the identification of 2000 genes of high cell-to-cell variation by using the function “FindVariableFeatures”. For multi-sample integration, we employed the function “FindIntegrationAnchors” to obtain “anchors” across individual samples. By inputting the anchors into the function “IntegrateData” and regressing out the influence of library size, percentage of mitochondria genes and cell-cycle scores, we created a “batch-corrected” expression matrix of all cells on the 2000 highly-variable genes. Based on the batch-corrected data, we performed Principal Component Analysis (PCA) with top 2000 variable features by using the function “RunPCA”. Cells were then clustered using the functions “FindNeighbors” and “FindClusters” with the first 50 principal components (PCs). Finally, we conducted nonlinear dimensional reduction for data visualization. In brief, UMAP was performed on the top 50 PCs by using the function “RunUMAP”.

### Cell type annotation and cluster marker identification

The function “FindAllMarkers” function was used to find markers for each of the identified clusters and annotated on the basis of the expression of canonical markers of particular cell types and the annotation reference created by SingleR (version 1.2.4) [71] based on the published single-cell transcriptome data of kidney cancer [22].

### Differential gene expression analysis

The parameter MAST in the function “FindAllMarkers” was used to perform differential gene expression analysis. A gene was considered significantly different with adjusted *p* < 0.05 [72].

### CNV estimation, identification of malignant cells and malignant cell subset analysis

To infer CNV patterns from the scRNA-seq data, we used an approach described on the website tutorial (https://github.com/broadinstitute/inferCNV). The identification of malignant cells was performed based on previous reports [44, 73]. Endothelial cells and myofibroblasts were considered references. And 30% of them (as spike-in cells) were randomly selected together with epithelial cells for CNV inference and hierarchical clustering in each patient. We considered the epithelial cells that clustered together with spike-in control cells to be ‘CNV-low’ cells, whereas the remaining cells were considered ‘CNV-high’, as malignant cells for further analysis.

### Diversity score

We calculated the diversity score of a tumor based on the gene expression profiles of malignant cells according to a published literature from Ma et al. [74]. The PCA analysis was performed to project the original expression profiles of all malignant cells to reducing the dimensionality of such datasets and to derive PCs, which could increase interpretability but at the same time minimize information loss. Then we calculated tumor diversity score to measure the degree of intratumoral heterogeneity based on the PCs within tumor by referring to the diversity score algorithms.

### Gene set enrichment analysis

We applied gene set enrichment analysis (GSEA) to analyze different pathways in different subclusters on the hallmark pathways and GO terms documented in the molecular signature database [75–77]. We applied the GSVA R package (version 1.38.0) to estimate pathway activity scores for single cell [78]. The differential activities of pathways were calculated using the limma R package (version 3.46.0) [79].

### Defining cell state scores

We used cell scores to evaluate the degree to which individual cell expressed a certain predefined expression gene set. To define progenitor exhausted and terminally exhausted phenotypes, we used the function “AddModuleScore” to calculate cell scores by using well-defined progenitor exhausted and terminally exhausted CD8^+^ T cell signatures [38, 61].

### Pseudotime trajectory analysis

To characterize the potential process of CD8^+^ T functional changes and determine the potential lineage differentiation, we applied the Monocle2 (version 2.16.0) R package [80] excluding MAIT cells. The gene-cell matrix of UMI counts was provided as the input to Monocle, and then, the newCellDataSet function was employed to create a CellDataSet with the parameter expressionFamily = negbinomial.size. We then performed the differentialGeneTest function to identify significantly different genes over time.

### Investigating genes correlated with certain cellular abundances

We estimated the abundance of each cell type according to the average expression levels of the top 50 cell-specific marker genes with average expression > 2 in each cell type in the bulk expression profiles. In addition, we manually excluded genes were obviously expressed in many other cell types [57]. Then, we calculated correlations between each gene and the abundance of certain cell types by using the function “corr” in the validation data. Furthermore, we identified different expression genes (DEGs) between two indicated cell types from single-cell profiles with log 2-transformed expression ratios > 1.5 or < − 1.5 and compared the relative correlation of the abundance of myofibroblasts or ECs associated with extracellular matrix remodeling-related genes between PTs and TTs.

### Quantification and statistical analysis

We performed all the statistical analyses using R software (version 4.0.0). For comparison of the signature scores or CIBERSORTx-inferred immune and nonimmune fractions between different cell groups and bulk RNA-Seq sample groups, a two-sided Wilcoxon test was used. Detailed descriptions of the statistical tests performed for individual analysis are provided in the Figure legends and Methods.

## Supplementary Information

**Additional file 1.** Supplementary Figures S1-S6 and corresponding legends.

**Additional file 2: Table S1**. Clinical variables for samples and patients.

**Additional file 3: Table S2.** Differential expression results for all lymphoid clusters, differential expression results for CD8^+^ T cell clusters, CD8-C1 related gene signatures, and progenitor/terminally exhausted signature scores for all CD8^+^ T cells, related to Fig. 2.

**Additional file 4: Table S3.** Differential expression results for all myeloid cell clusters, for PT and TT from all TAMs clusters, and published gene signatures for GSEA, related to Fig. 3.

**Additional file 5: Table S4.** Differential expression results for all tumor cell clusters, for PT and TT from all tumor clusters, related to Fig. 4.

**Additional file 6: Table S5.** Differential expression results for all endothelial clusters and all myofibroblast clusters, and published gene signatures for GSEA, related to Fig. 5.

**Additional file 7: Table S6.** Ligand-receptor means and *p*-values for all cell interactions in ART, PT and TT (from CellPhoneDB v2.0) and created gene signatures, related to Fig. 6.

**Additional file 8.** Review history.

## Data Availability

The raw single cell RNA sequencing data has been deposited in the Genome Sequence Archive in National Genomics Data Center, China National Center for Bioinformation / Beijing Institute of Genomics, Chinese Academy of Sciences. The accession number for the sequencing data reported in this paper is HRA000963 at https://ngdc.cncb.ac.cn/gsa-human [81]. For the validation cohort with PT and paired TT, raw bulk RNA-seq data were obtained from the database of NCBI Sequence Read Archive (SRA) under the accession code PRJNA596338 (https://www.ncbi.nlm.nih.gov/bioproject/PRJNA596338) [82]. For the CheckMate 025 cohort, normalized bulk RNA-seq and clinical data were obtained from published results [19, 20]. For TCGA-KIRC cohort, preprocessed bulk RNA-seq data were downloaded from UCSC Xena (https://xena.ucsc.edu/). For the IMmotion150 cohort, the datasets are available from the European Genome-Phenome Archive (EGA) repository, the accession number is EGAS00001002928 (https://ega-archive.org/studies/EGAS00001002928) [83]. All code used to analyze data are available on GitHub, at https://github.com/zhangqi234/RCC-tumor-thrombus-scRNA.
