# Supplementary material for: Decoding the multicellular ecosystem of vena caval tumor thrombus in clear cell renal cell carcinoma by single-cell RNA sequencing
Source: Genome Biol. 2022 Mar 31;23:87. doi: 10.1186/s13059-022-02651-9 (PMC8969307; doi:10.1186/s13059-022-02651-9)
Supplement: Supplementary file 1 — Additional file 1. Supplementary Figures S1-S6 and corresponding legends. [file 13059_2022_2651_MOESM1_ESM.docx]

Supplementary Information for

**Decoding the multicellular ecosystem of vena caval tumor thrombus in clear cell renal cell carcinoma by single-cell RNA sequencing**

***This file includes:***

**Additional file 1: Supplementary Figures**

**Fig. S1** Quality control of single-cell sequencing data, related to Fig.1

**Fig. S2** T and NK cell subtypes in ARTs, PTs and TTs, related to Fig.2

**Fig. S3** Myeloid cell subtypes in ARTs, PTs and TTs, related to Fig.3

**Fig. S4** Characteristics of malignant cells in PTs and TTs, related to Fig.4

**Fig. S5** Endothelial and myofibroblast subtypes in ARTs, PTs and TTs, related to Fig.5

**Fig. S6** Expression models of ligand and receptor genes in different cell types and potential intercellular interactions between ARTs, PTs and TTs, related to Fig.6

**Supplementary Figures 1-6**


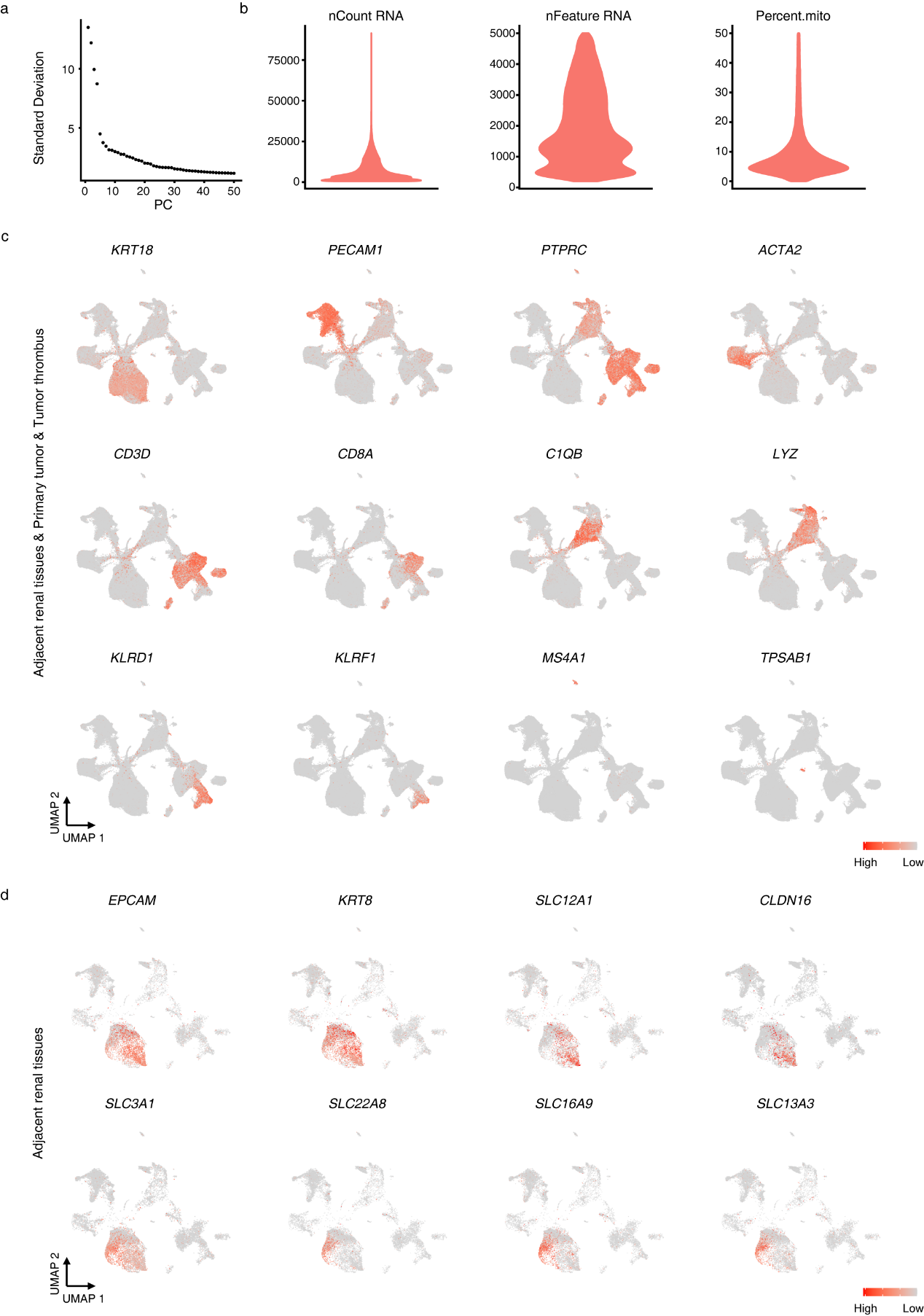


**Fig. S1** Quality control of single-cell sequencing data, related to Fig.1

**a** Scree plot showing the top 50 PCs of principal component analysis, and all of them were used in the downstream analysis.

**b** Violin plots showing the number of UMIs (nCount), number of genes (nFeature) detected and percent of mitochondrial-derived transcripts (percent.mito) per single cell after quality control.

**c** UMAP plots showing the expression levels of marker genes defined for the cell types in ARTs, PTs and TTs.

**d** UMAP plots showing the expression levels of marker genes defined for epithelial cell, proximal tubule and loop of Henle in ARTs.


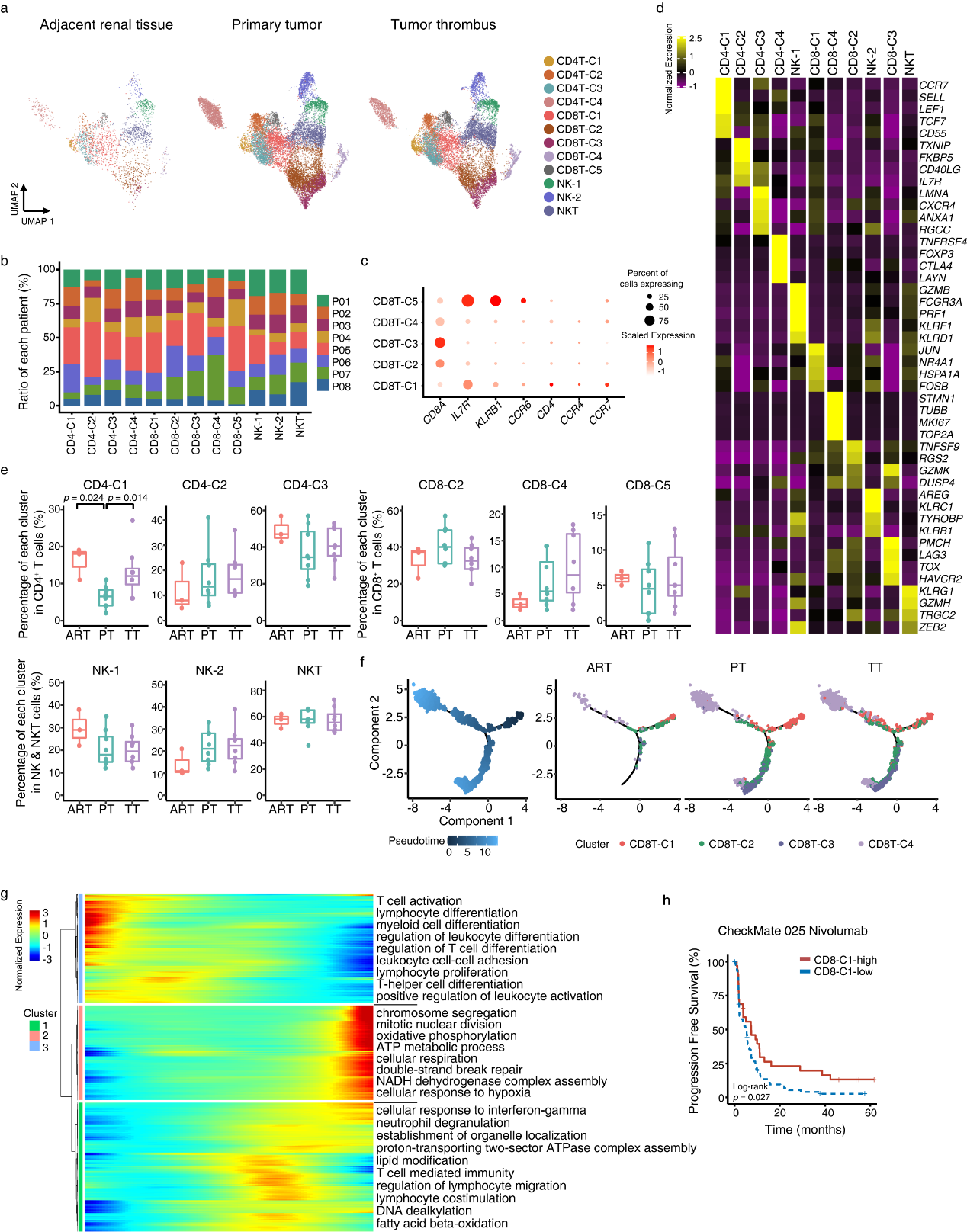


**Fig. S2** T and NK subtypes in ARTs, PTs and TTs, related to Fig.2

**a** UMAP plots representing T/NK cells derived from ARTs, PTs and TTs.

**b** Bar plot illustrating the distribution of each patient in each T/NK subtype. Each bar corresponds to one cell type cluster, colored according to sample name.

**c** Dot plot representing the expression of MAIT T cell marker genes in each subcluster of CD8^+^ T cells.

**d** Heatmap showing the expression of marker genes in each subtype of T/NK cells.

**e** Box plots illustrating the fraction of T/NK subgroups in ARTs, PTs and TTs. *p* values were determined by a two-sided Wilcoxon test.

**f** Differential pseudotime trajectory analysis of CD8^+^ T cells from ART, PT and TT samples. CD8^+^ T cells subtypes are labeled by colors.

**g** Heatmap showing the dynamic changes in gene expression over pseudotime. The GO pathways of the differentially expressed genes are listed on the right.

**h** Kaplan-Meier plot showing that patients from the nivolumab arm of the CheckMate 025 cohort with a high CD8-C1 signature were associated with improved progression-free survival (PFS).


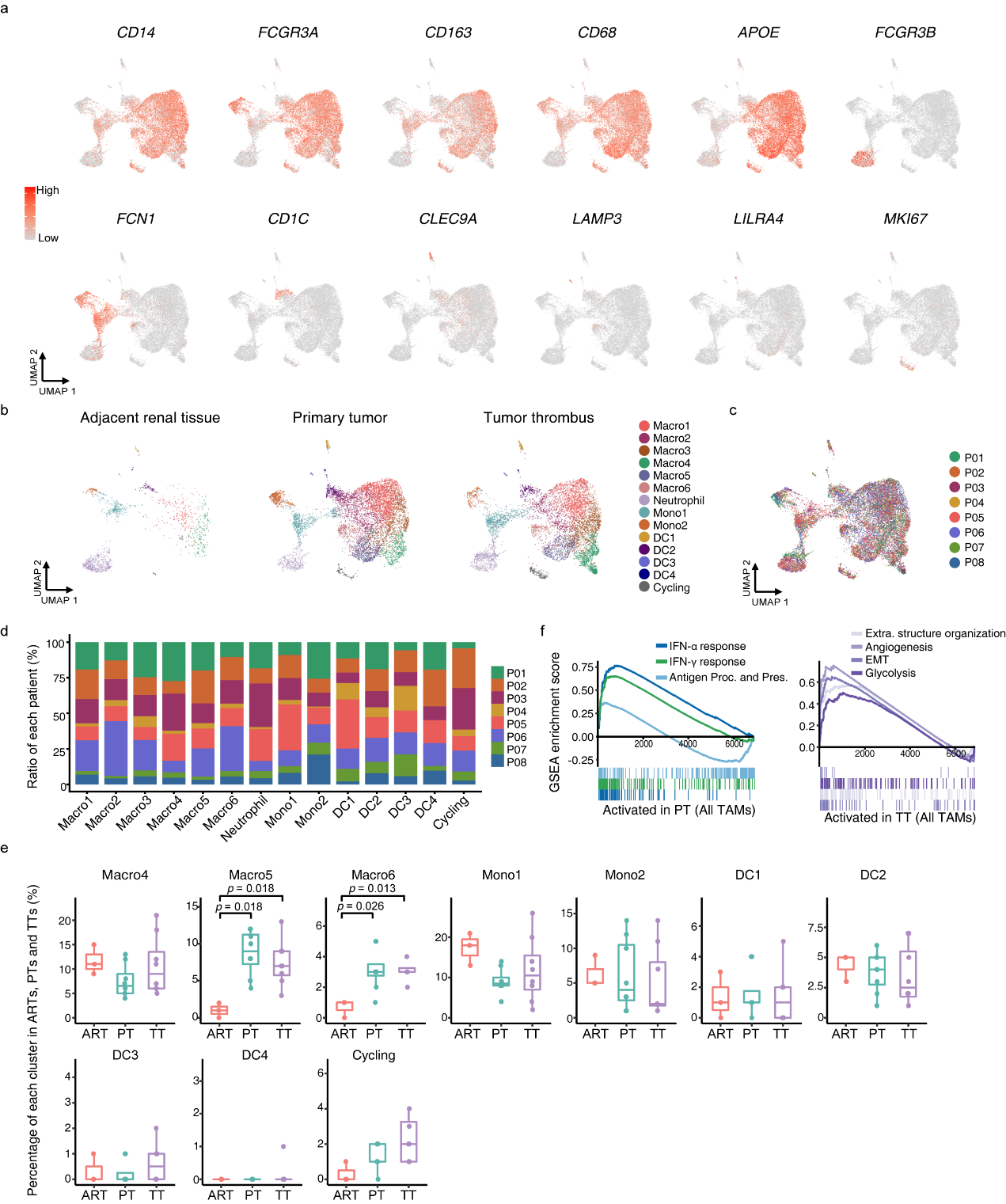


**Fig. S3** Myeloid cell subtypes in ARTs, PTs and TTs, related to Fig.3

**a** UMAP plots showing the expression of selected marker genes for the defined cell types.

**b** UMAP plots representing myeloid cells derived from ARTs, PTs and TTs.

**c** UMAP plot illustrating myeloid cells, clustered and color-coded, according to patient.

**d** Bar plot illustrating the distribution of each patient in each myeloid subtype. Each bar corresponds to one cell type cluster, colored according to sample name.

**e** Box plots illustrating the fraction of myeloid subgroups in ARTs, PTs and TTs. *p* values were determined by a two-sided Wilcoxon test.

**f** GSEA of interferon response and antigen processing and presentation enrichment scores in all TAMs of PTs compared with those of TTs, Extra. structure organization, extracellular structure organization, angiogenesis, EMT and glycolysis enrichment scores in all TAMs of TTs compared with those of PTs.


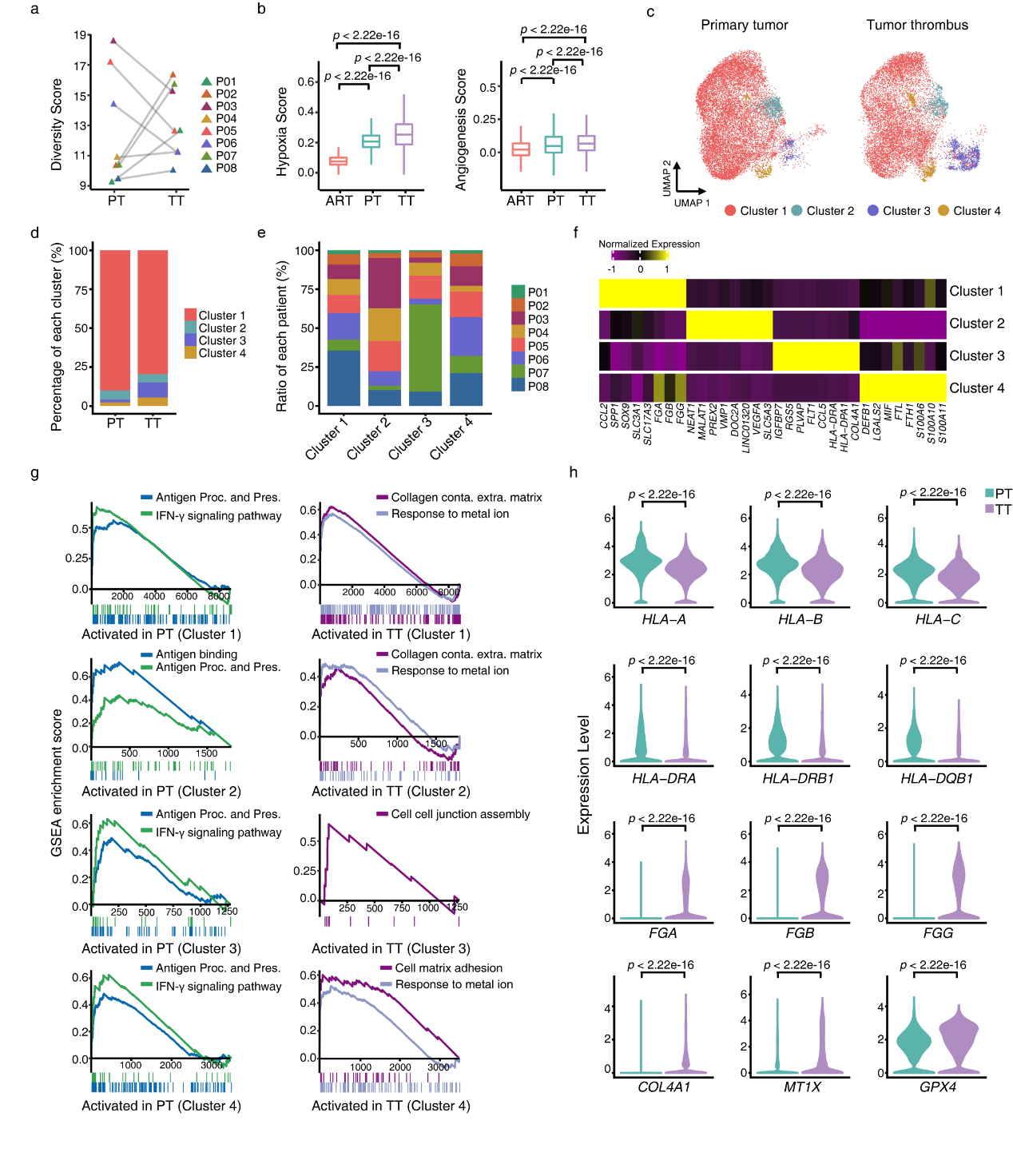


**Fig. S4** Characteristics of malignant cells in PTs and TTs, related to Fig.4

**a** Diversity scores of the malignant cells of PTs and TTs in each patient.

**b** Box plots showing the hypoxia and angiogenesis scores of proximal tubule cells from ART, and malignant cells from PT and TT samples. *p* values were determined by a two-sided Wilcoxon test.

**c** UMAP plots representing malignant epithelial cells derived from PTs and TTs.

**d** Bar plot illustrating the fraction of malignant cell subsets in PTs and TTs.

**e** Bar plot representing the distribution of each patient in each malignant cell subtype. Each bar corresponds to the cell type cluster, colored according to the sample name.

**f** Heatmap showing the expression of marker genes in each subtype of malignant cells.

**g** GSEA of the IFN-γ signaling pathway or antigen presentation and processing (Antigen Proc. and Pres.) related pathways enrichment scores in clusters 1-4 of PT malignant cells compared with TT cells; extracellular matrix organization related pathways or response to metal ion enrichment scores in clusters 1-4 of TT malignant cells compared with PT ones.

**h** Violin plots representing the expression level of antigen presentation and processing-, extracellular matrix organization- and metal ion binding-related differentially expressed genes between PTs and TTs. *p* values were determined by a two-sided Wilcoxon test.


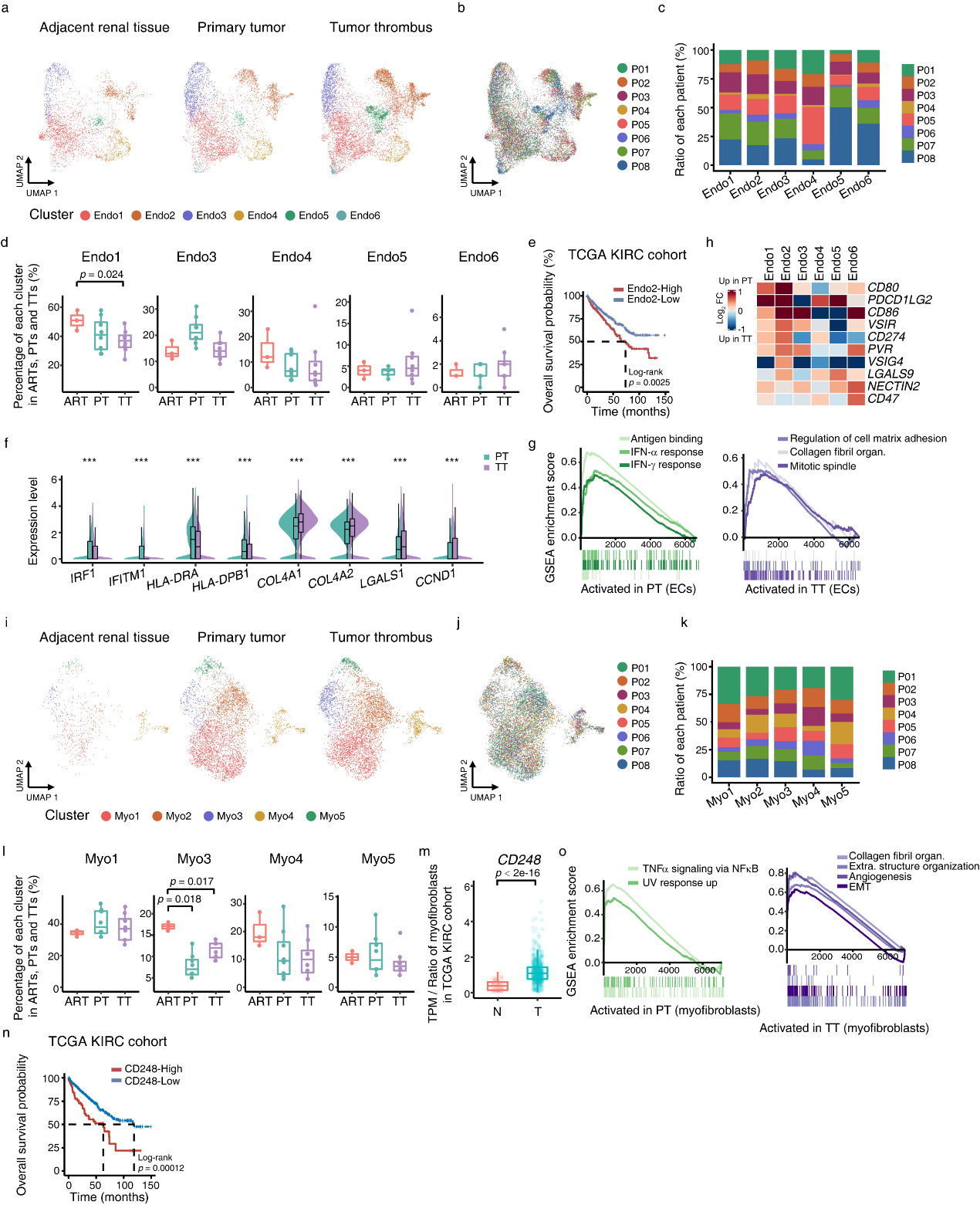


**Fig. S5** Endothelial and myofibroblast subtypes in ARTs, PTs and TTs, related to Fig.5

**a** UMAP plots representing endothelial cells derived from ARTs, PTs and TTs.

**b** UMAP plot illustrating endothelial cells, clustered and color-coded, according to patient.

**c** Bar plot illustrating the distribution of each patient in each endothelial subtype. Each bar corresponds to one cell type cluster, colored according to the sample name.

**d** Box plots illustrating the fraction of endothelial subgroups (Endo1, Endo3, Endo4, Endo5 and Endo6) in ARTs, PTs and TTs. *p* values were determined by a two-sided Wilcoxon test.

**e** Kaplan-Meier plot showing that KIRC patients in the TCGA dataset with high expression of Endo2 signature had shorter overall survival (OS). The high and low groups were divided based on the value of the mean expression level of each subtype gene set by using the “surv-cutpoint” function.

**f** Violin and box plots comparing the expression distributions of interferon and antigen presentation related genes and ECM remodeling and cell cycle-related genes between PTs and TTs. *p* values were determined by a two-sided Wilcoxon test. ***, *p* < 0.001.

**g** GSEA analysis of interferon response and antigen binding enrichment scores in all ECs of PTs compared with those of TTs, regulation of cell matrix adhesion, Collagen fibril organ., collagen fibril organization, and mitotic spindle enrichment scores in all ECs of TTs compared with those of PTs.

**h** Heatmap depicting the differential expression fold change of each subset of ECs in PTs compared with TTs for immune checkpoint and evasion-related genes.

**i** UMAP plots representing myofibroblasts derived from ARTs, PTs and TTs.

**j** UMAP plot illustrating myofibroblasts, clustered and color-coded, according to patient.

**k** Bar plot illustrating the distribution of each patient in each myofibroblast subtype. Each bar corresponds to one cell type cluster, colored according to sample name.

**l** Box plots illustrating the fraction of myofibroblast subgroups (Myo1, Myo3, Myo4 and Myo5) in ARTs, PTs and TTs. *p* values were determined by a two-sided Wilcoxon test.

**m** Box plot showing the expression level of *CD248* in all myofibroblasts between the normal (N) and tumor (T) samples in the TCGA KIRC cohort. *p* values were determined by a two-sided Wilcoxon test.

**n** Kaplan-Meier plot showing that KIRC patients in the TCGA dataset with high expression of *CD248* had worse overall survival.

**o** GSEA analysis of TNF-α signaling via NFκB and UV response up enrichment scores in all myofibroblasts of PTs compared with those of TTs, Collagen fibril organ., collagen fibril organization, Extra. structure organization, extracellular structure organization, angiogenesis and EMT enrichment scores in all ECs of TTs compared with those of PTs.


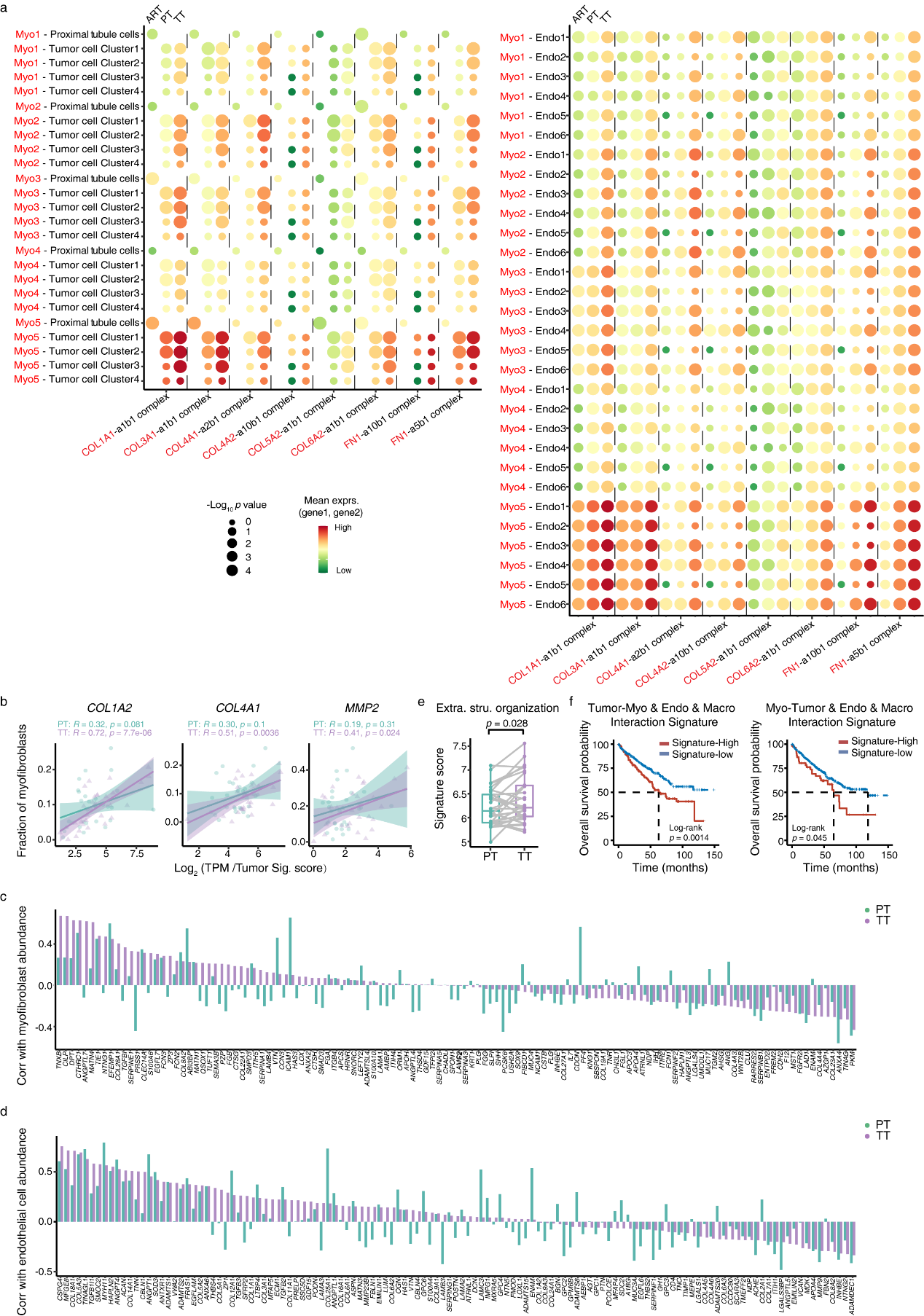


**Fig. S6** Expression models of ligand and receptor genes in different cell types and potential intercellular interactions between PTs and TTs, related to Fig.6

**a** Dot plot showing inferred interactions between the subclusters of myofibroblasts, epithelial cells (malignant cells from PTs and TTs and proximal tubule cells from ARTs) and endothelial cells. Circle size indicates the significance of the interaction, and circle color indicates the mean expression of ligand and receptor genes. The red letters represent ligands, and the black letters represent receptors.

**b** Scatterplots representing the correlation between member genes of the extracellular matrix assembly, including *COL1A2, COL4A1* and *MMP2,* and the myofibroblast ratio in the PTs and TTs of the validation cohort. The Pearson coefficient (R) and associated *p* value are reported for each correlation.

**c** Bar plot representing the correlation between extracellular matrix remodeling-related genes and myofibroblast relative abundance involved in PTs and TTs in detail, which showed genes with log2 foldchange (FC) <-1.5.

**d** Bar plot showing the correlation between extracellular matrix remodeling-related genes and endothelial cell relative abundance involved in PTs and TTs in detail, which showed genes with log2 FC < -1.5.

**e** Signature score for extracellular structure organization in paired PT-TT bulk RNA-seq samples in the validation cohort. *p* values were determined by a two-sided Wilcoxon test. Extra. stru. organization, extracellular structure organization.

**f** Overall survival for the TCGA KIRC cohort based on high tumor-stroma and stroma-tumor interaction signatures versus low signatures.
