# Supplementary material for: Decoding the multicellular ecosystem of vena caval tumor thrombus in clear cell renal cell carcinoma by single-cell RNA sequencing
Source: Genome Biol. 2022 Mar 31;23:87. doi: 10.1186/s13059-022-02651-9 (PMC8969307; doi:10.1186/s13059-022-02651-9)
Supplement: Supplementary file 8 — Additional file 8. Review history. [file 13059_2022_2651_MOESM8_ESM.docx]

Review History

**First round of review**

**Reviewer 1**

**Are you able to assess all statistics in the manuscript, including the appropriateness of statistical tests used?**

**Yes**

**Comments to author:**

Yue Shi and author present "Decoding the multicellular ecosystem of vena caval tumor thrombus in renal cell carcinoma by single-cell RNA sequencing." Overall, this is an important resource generated to help shed light on the differences between the primary tumor and the tumor thrombus (and its correlative microenvironment). However, the authors present a significant amount of data which is difficult to understand as the figures/supp materials require significant amounts of time to interpret and don't entirely follow the text. There seems to be an opportunity to take these 6 figures with many subpanels and simplify this vast amount of data given the conclusions.

Major:
1) The authors mention that they use validation cohorts including CheckMate025, KIRC TCGA, and IMmotion150. Most if not all these patients are clear cell RCC patients. Why then do they include at TFE3 RCC sample in their data set? It is combined in many cases and it leaves confusion in the overall interpretation of data. It would seem important given the populations of bulk RNAseq that are being used for validation to not include the TFE3 RCC sample.
2) There are several strong statements (e.g. lines 204-206) which seems to be difficult to justify. In this case, were these CDC8-C1 signatures identified in the CheckMate025 based on patients with tumor thrombi? Were there other features from the CheckMate025 cohort that could have contributed?
3) The use of tSNE for scRNAseq papers is somewhat outdated. Can the authors comment why they chose this over UMAP?
4) In Figure 3e, the authors state that the CD8-C1 cells are increased in TTs compared with PTs but these are still both lower than in ARTs. Can the authors clarify?
5) Lines 338-340 need further explanation as it unclear how this analysis was performed
6) Can the authors comment how tumor thrombi are related with pulmonary metastases based on their findings? Would be good to mention in discussion.

Minor:
1. There are typographical errors throughout the figures. E.g. Figure 1b, "Paired" is misspelled.
2. Figure 1g doesn't describe what N vs C vs T stands for.
3. What are these validation cohorts 1,2,3,4 and in general the figure legends are very short with little explanation requiring a lot of work by a reader

**Reviewer 2**

**Are you able to assess all statistics in the manuscript, including the appropriateness of statistical tests used?**

**Yes**

**Comments to author:**

Shi et al perform scRNA on a cohort of RCC patients with both primary tumor and thrombus. Ultimately the paper shows no major differences between the the TT and PT for many of the clusters but did find enrichment for progenitor exhausted T cell, and ECM remodelling TAM phenotype. Strenghts inlcude a valdiation cohort of 40 paired RNAseq samples of tumor and thrombus but there are several practical limitations.


Fundamentally the concern is related to the apparent lack of batch correction between the samples and pairs. I could not emphasize more about the importance of this in analyzing pooling samples since it could change the results profoundly. There are a number of possible hints of batch bias in, for example, epithelial and endothelium cell clustering results. Some clusters tend to be enriched by a specific patient. Of course, it's very possible it's due to patient/sample type heterogeneity but it's hard to tell without some batch correction measures applied. Given the importance of the difference being interpreted by the authors between patients and sampling sites, it's really a key question to be addressed. Some specific comments are:
1. How are the "malignant cells" mentioned on page 7 being determined?
> 2. There seems "under-clustering" existing in, at least, epithelial and endothelium cell clustering results. It's obvious that there could be more clusters to be called. What's the justification of not doing so? Could this be the reason of finding both glycolysis and OXPHOS signal enrichment in the cluster 1 of epithelial cells?
> 3. What's the justification of using very loosen filters, 200<= number of genes detected <= 5000 and percent of mitochondrial UMI <= 50% (page 23), in filtering out bad QC cells? It is common to use 500 genes and 20% MT reads as the cutoff for human data and there's study suggests more stringent mitochondrial read % cutoff for human. Won't the looser filters bring in more bad QC cells which increases noise?
> 4. GSVA/GSEA analysis were developed originally for bulk RNAseq whole transcriptome analysis. Using them in the scRNAseq space is uncommon, how can we be assured about the possible impact of the much lower number of genes detected in each cell (as low as 200) and the gene dropouts is not biasing the enrichment analysis results?
> 5. To what extent the tumor thrombus results could be influenced by the direct contact with the peripheral circulation? Isn't it reasonable to think the higher number of more naïve progenitor-exhausted CD8 cells (cluster 1) as well as CD4 cluster 1 is the work of higher blood infiltration? Given the hypoxia environment in TT and PT as compared to ART, isn't it reasonable to see higher exhausted cells, CD8 C3 & C4 for example, in them?
6. There are no mulitregional profiling performed and previous effort from TracerRX cohort showed that the TT can be seeded in different mechanism (ie early event vs advance subclones). Can the authors utilize either their discovery or validation cohort to comment on ITH and the TME with both the PT and TT.> 7. In CellPhoneDB analysis, how do the authors justify doing analysis between only major cell types given so many clusters existing within each cell type? And how can we interpret the results from pooling cells with different phenotype or functional state?
> 8. Is it reasonable approach to use the top 50 DEG genes (page 25) for building the signature of a specific cell cluster/function state without any filtering? How do the authors propose to avoid signal from other cell types which were not included in the DEG analysis? For example, immune related signature genes which are seen with signal from epithelial cells?
> 9. The parameter MAST (hurdle model) in the FindAllMarkers function was used to perform DEG analyses (page 24) which I believe is not taking into account of possible batch effect from patients and sample types. The author should consider consider using "negbinom" or "LR" parameter which allow incorporating the patient and the sampling site (ART, PT or TT) as a covariate in the DEG model?

**Authors Response**

**Point-by-point responses to the reviewers’ comments:**

**Reviewer 1**

1) The authors mention that they use validation cohorts including CheckMate025, KIRC TCGA, and IMmotion150. Most if not all these patients are clear cell RCC patients. Why then do they include at TFE3 RCC sample in their data set? It is combined in many cases and it leaves confusion in the overall interpretation of data. It would seem important given the populations of bulk RNAseq that are being used for validation to not include the TFE3 RCC sample.

*Response: We appreciate your great suggestion. According the reviewer’s suggestion, we have removed the data of TFE3 RCC sample from CIBERSORTx analysis, and re-evaluated the proportion of relevant subgroups in the corresponding validation cohorts in our study. The results are consistent with what we found before. Furthermore, we have updated all the figures that related to CIBERSORTx analysis (Fig. 3h, Fig. 5f, 5l, 5m, Fig. 6b, Fig. S5k and Fig. S6b), and added this description in the main methods section of the revised manuscript (page 27, line 576).*

2) There are several strong statements (e.g. lines 204-206) which seems to be difficult to justify. In this case, were these CDC8-C1 signatures identified in the CheckMate025 based on patients with tumor thrombi? Were there other features from the CheckMate025 cohort that could have contributed?

*Response: Thanks for your great question. We are very sorry for missing this description. And there is no information about patients with or without tumor thrombi in CheckMate025 cohort. Studies have found that tissue-resident CD8+ T cells are involved in tumor immunotherapy, and often associated with improved patient outcomes [PMID: 33755718; PMID: 31255505]. Recently, one study about RCC also found that the signature of tissue-resident CD8+ T cells predict better progression free survival (PFS) with immune checkpoint blockade (ICB) therapy [PMID: 33861994]. Since we don’t have the survival information of 30 RCC patients with tumor thrombi cohort, for validate whether CD8-C1 cells have a better response to anti-PD-1 therapy, we used the CheckMate025 cohort with nivolumab (anti-PD-1) treatment, which is a clinical phase III study of advanced renal cell carcinoma patients. By analyzing the data of this cohort, we found the high levels of the CD8-C1 signature were associated with improved PFS (Additional file 1: Fig.S3h), which means CD8-C1 cells positively correlates with an improved duration of response to checkpoint blockade therapy. To avoid misunderstanding, we modified this sentence as “Although there is no information about patients with or without tumor thrombi in CheckMate 025 cohort, combined with our new discovery that TTs has a high level of CD8-C1 cells, we suggest that samples like tumor thrombi with a high level of tissue-resident CD8+ T cells may indicate a good response to anti-PD-1 immune therapy in RCC patients. However, it is necessary for further clinical and mechanistic investigation in regard to the associations between tissue-resident CD8+ T cells with ICB response in RCC patients with TTs given the small sample size of our single-cell and validation cohorts.” in the revised manuscript (page 11, line 225-232). In addition, that research revealed there is a continuous pseudotime trajectory from tissue-resident T cells to NK-like T cells in the patients with complete response and mixed response to ICB by TCR trajectory analysis [PMID: 33861994]. But the resistant and untreated patients displayed no such trajectory [PMID: 33861994]. These results suggest that such transformation may be characteristic of response to ICB [PMID: 33861994]. Furthermore, we found that there is a cluster named “NKT cell” in our study that is very similar to the NK-like T cells in the published papers both with high expression of NK cell and CD8+ T cell markers (e.g., CD8A, CD8B, KLRD1 and KLRF1), and the high level of NKT cell signature also have a better response to anti-PD-1 therapy in CheckMate025 cohort (Response Fig1). So, we speculated that the level of NK-like T cells might be another feature of CheckMate025 cohort that contributed to anti-PD-1 therapy.*

*Response Fig1. Kaplan-Meier plot showing that patients from the nivolumab arm of the CheckMate 025 cohort with a high NKT signature were associated with improved progression-free survival.*

*Moreover, one study published recently [PMID: 33219016], showed that by using immunofluorescence staining, high levels of CD8+ tumor-infiltrating cells (TICs) expressing PD-1 but not TIM-3 and LAG-3 predicted response to nivolumab in CheckMate025 cohort. The tumors with high CD8+ PD-1 + TIM-3 - LAG3- TICs were enriched for several inflammatory pathways, immune-related gene signature scores and immune cell populations such as NK activated T cells, plasma cells and helper T cells, likely representing the inflamed tumors harboring mildly exhausted tumor-specific T cells, which can be re-activated by anti-PD-1 therapy [PMID: 33219016]. So, the CD8+ TIC immunophenotyping might be another feature of CheckMate025 cohort to predict response to nivolumab.*

3) The use of tSNE for scRNAseq papers is somewhat outdated. Can the authors comment why they chose this over UMAP?

*Response: We appreciate your great comment. According to the reviewer’s suggestion, we updated the UMAP plots instead of the tSNE plots for data visualization and modified the description in the method section in the revised manuscript. In addition, we noticed that especially in Fig. 1c in the revised manuscript, UMAP was better than tSNE in the presentation of relatedness of global structure of each cell type without splitting T cells and myeloid cells.*

4) In Figure 3e, the authors state that the CD8-C1 cells are increased in TTs compared with PTs but these are still both lower than in ARTs. Can the authors clarify?

*Response: Thanks for your great question. Tissue resident CD8+ T cells has been found a lot in normal kidney tissues [PMID: 33535505; PMID: 32973321; PMID: 29093030]. But the present exploratory study did not elucidate the mechanisms related to the high abundance of T cells within human kidneys including the tissue resident CD8+ T cells [PMID: 32973321]. The tough renal metabolic environment including the toxins of the urine and dead parenchymal cells or debris from tubules and glomeruli might be a potential impact for tissue resident CD8+ T cells residing. Moreover, the adjacent renal tissues are not truly “normal”, and inflammatory response-related pathways like allograft rejection, IFN-gamma response and IFN-alpha response, are generally enriched in normal tissue adjacent to the tumor (NAT) in most tissue types [PMID: 29057876]. That might be another hint that tissue resident CD8+ T cells are abundant in tissues adjacent to tumor for immunosurveillance. In addition, by using single RNA-seq analysis, a study about hepatocellular carcinoma [PMID: 33357445] found that the ratios of tissue resident CD8+ T cells are significant differences between non-tumor tissues and tumors. A larger proportion of tissue resident CD8+ T cells was observed in adjacent non-tumor tissues rather than in tumors [PMID: 33357445], which is consistent with our finding in RCC. According the reviewer’s question, we added this information and the relevant references in the revised manuscript (page 8-9, line 176-183).*

5) Lines 338-340 need further explanation as it unclear how this analysis was performed.

*Response: We appreciate your great suggestions. According to the published papers to resolve the composition of the PT and TT microenvironment [PMID: 32901110; PMID: 27124452], we first calculated the relative abundance of each cell type in the bulk RNA expression profiles, according to the average expression levels of the top 50 cell-specific marker genes excluding those that were obviously expressed in many other cell types. Then, we estimated pearson correlations between each gene and the relative abundance of certain cell types by using the corr function in the validation cohort. Furthermore, we identified differential expressed genes (DEGs) between two indicated cell types from single-cell profiles with log 2-fold change cutoff of +/-1.5, and highlighted some DEGs with high correlation in PTs and TTs in Fig. 6c and Fig.6e in the revised manuscript. For comparing the correlation of the myofibroblasts or ECs abundance related with ECM-related genes between PTs and TTs, box plots are used to show the correlation scores between both of them. In addition, we have modified the description of this analysis in the main text and methods in the revised manuscript (page 17-18, line 373-379, page 26 line 565-572).*

6) Can the authors comment how tumor thrombi are related with pulmonary metastases based on their findings? Would be good to mention in discussion.

*Response: Thank you so much for your great suggestion. According to the reviewer’s suggestion, we added the related information in the revised manuscript. (page 20, line 431-441). Unlike the kidney primary tumors, tumor thrombi form from contiguous tumor growth into the renal vein or inferior vena cava, that means the cancer cells have intravasated into vein, and might have more opportunity to spread to lung. But the malignant cells of TTs are not independently suspended in the vasculature, they form a special structure with surrounding immune cells and stroma cells. In our study, we found the extracellular matrix (ECM) remodeling related pathways are enhanced in TTs, which suggests that they are associated with tumor invasion and growth. Some studies [PMID: 9105051; PMID: 27739043] demonstrated the ECM is important at initial stages of metastasis, where interactions between tumor cells and extracellular matrix induced an invasive phenotype. Although there is no direct evidence that RCC patients with tumor thrombi are more likely to develop pulmonary metastasis, one paper [PMID: 34608135] published recently showed TT grade is an independent predictor of metastatic outcome irrespective of the grade of the PT, which suggests the invasion ability of tumor cells in TTs is very important for metastatic potential. Thus, targeting the influence factors of invasion such as ECM remodeling associated genes could be effective for reducing metastases. In addition, we have added this discussion in the main text in the revised manuscript (page 20, line 430-440).*

Minor comments:

There are typographical errors throughout the figures. E.g. Figure 1b, "Paired" is misspelled.

*Response: We are deeply sorry for this error. We have fixed the mistake in Figure 1b in the revised manuscript.*

Figure 1g doesn't describe what N vs C vs T stands for.

*Response: We are deeply sorry for missing this information. We have added the information in the figure legend of Figure 1g in the revised manuscript. (page 30, line 655).*

What are these validation cohorts 1,2,3,4 and in general the figure legends are very short with little explanation requiring a lot of work by a reader.

*Response: We are deeply sorry for missing this information. We have added the number in Figure 1b and the detailed cohort information in the figure legend of Figure 1b in the revised manuscript. (page29-30, line 645-649)*

**Reviewer 2**:

1. How are the "malignant cells" mentioned on page 7 being determined?

*Response: Thanks for your great question. According the reviewer’s suggestion, we added detailed information of how to determine malignant cells and cited relevant references in methods in the revised manuscript. (page 25, line 530-533). Actually, we use inferCNV (R package) to identify the malignant epithelial cells. According to the published papers [PMID: 32822576; PMID: 32385277], endothelial cells and myofibroblasts were randomly selected as reference controls, and 30% of them (as spike-in cells) were randomly selected together with epithelial cells for CNV inference and hierarchical clustering in each patient. The epithelial cells excluding clustered with endothelial cells and myofibroblasts are considered to obtain large CNV aberrations and determined as malignant cells for further analysis.*

2. There seems "under-clustering" existing in, at least, epithelial and endothelium cell clustering results. It's obvious that there could be more clusters to be called. What's the justification of not doing so? Could this be the reason of finding both glycolysis and OXPHOS signal enrichment in the cluster 1 of epithelial cells?

*Response: Thank you so much for your great suggestions. According to the reviewer suggestion, we re-clustered the malignant cells and endothelial cells. For malignant cells, we tried several different clustering parameters and calculated the scores of differentially expressed pathways by GSVA according to the analysis in Fig. 2f in the revised manuscript. For example, we re-clustered the malignant cells to 6 clusters, and both glycolysis and oxidative phosphorylation pathways are still enhanced in cluster 1 (Response Fig. 1A). Then, we tried to adjust the clustering parameters bigger, and got more detailed clusters. And we found that in cluster 3 separated from cluster 1, both of glycolysis and oxidative phosphorylation pathways are activated (Response Fig.1B). So, we think that in kidney cancer, both glycolysis and OXPHOS signal enrichment is existing. And this result is also consistent with another published single cell RNA-seq study of RCC [PMID: 33711272].*

*Response Fig1. Malignant cells in PTs and TTs re-clustered results. Differentially expressed pathways were scored per cell by GSVA among 6 malignant cell subclusters (A) and 9 malignant cell subclusters (B).*

*For endothelial cells, we re-clustered them in to 7 clusters, and found endothelial subtypes are more detailed. Meanwhile, the results are consistent with those in the submitted version. The new figures and results are updated in Fig. 5a-g in the revised manuscript (page 12-13, line 284-297, page 13, line 300-303 and line 306-309, page 36-37, 725-738), in the supplementary Fig. S5a-f in additional file 1. And the new additional file 6 table S5 is also updated.*

3. What's the justification of using very loosen filters, 200<= number of genes detected <= 5000 and percent of mitochondrial UMI <= 50% (page 23), in filtering out bad QC cells? It is common to use 500 genes and 20% MT reads as the cutoff for human data and there's study suggests more stringent mitochondrial read % cutoff for human. Won't the looser filters bring in more bad QC cells which increases noise?

*Response: We appreciate your great suggestions. We agree with the reviewer about the stringent thresholds for filtering low quality cells for most of single cell RNA-seq data. According the reviewer’s suggestion, we tried many different thresholds for filtering including the threshold of 500 genes and 20% MT reads asthe cutoff. We found that if we use the stringent thresholds for filtering, the group of malignant cells lost about 30% and the percent of myeloid cells reduced about 20% compared with current filtering parameters in our manuscript. That would be a big loss for further analysis, especially for malignant cells. In addition, the method of one study [PMID: 32107344] related to human normal kidney pointed out that a cutoff of < 50% mitochondrial reads per cell was suitable for the data from the version 3 kit of single RNA-seq 10× Genomics library construction which is what we used in our research. It has been observed by them and other groups that, for certain tissues, including kidney, 10× Genomics version 3 chemistry results in a higher percentage of mitochondrial reads, irrespective of the viability status of the cells [PMID: 32107344]. So, we used percent of mitochondrial UMI <= 50% as our threshold. Furthermore, according to another paper [PMID: 31604275] mentioned that a threshold of 200 genes was retained to avoid eliminating neutrophils for the mature kidney immune cells, and other published papers [PMID: 32398875; PMID: 33504936; PMID: 32744436; PMID: 30093597] about single cell RNA-seq in which they all used “200 <= number of genes” as the threshold for filtering. Finally, we applied 200 <= number of genes detected <= 5000 and percent of mitochondrial UMI <= 50% for filtering low quality cells. In addition, we have cited these references in line 508-509, page 23.*

4. GSVA/GSEA analysis were developed originally for bulk RNAseq whole transcriptome analysis. Using them in the scRNAseq space is uncommon, how can we be assured about the possible impact of the much lower number of genes detected in each cell (as low as 200) and the gene dropouts is not biasing the enrichment analysis results?

*Response: We appreciate your great comments. We couldn't agree more that GSVA and GSEA analysis are ubiquitous tools used for exploring biologically meaningful information from gene expression data from both microarray and bulk RNA-seq experiments. And when we were analyzing our single cell RNA-seq data, we found a number of research articles about single cell transcriptomics used the method of GSVA/GSEA to analyze different biological pathways at single cell level [PMID: 33571124; PMID: 33711272; PMID: 33357445; PMID: 32396851; PMID: 33033240; PMID: 32351704; PMID: 33504936]. According to these published papers, we estimated the pathway activity of individual cells by applying GSVA/GSEA R package with standard settings at single cell level. Furthermore, we are very sorry that there is a mistake in Fig.2f and Fig.S4f without changing the results in the manuscript, so, the figures of Fig.2f and Fig.S4f are updated in the revised manuscript.*

5. To what extent the tumor thrombus results could be influenced by the direct contact with the peripheral circulation? Isn't it reasonable to think the higher number of more naïve progenitor-exhausted CD8 cells (cluster 1) as well as CD4 cluster 1 is the work of higher blood infiltration? Given the hypoxia environment in TT and PT as compared to ART, isn't it reasonable to see higher exhausted cells, CD8 C3 & C4 for example, in them?

*Response: Thanks for your good questions. It is reasonable to think the blood cells, especially immune cells in peripheral circulation have more opportunity to infiltrate into tumor thrombus, as tumor thrombus growth in the blood vessels can be fully contact with peripheral blood. For CD4+ cluster 1(CD4-C1) with high expression of CCR7, TCF7 and SELL as the naïve CD4+ cells, as expected, the proportion of them in TTs is higher than that in PTs (in Fig. S4e in the revise manuscript). And kidney serves as the key organ for blood filtration and circulation, the ratio of naïve CD4+ cells in ARTs is also higher than that in PTs (in Fig. S4e in the revise manuscript). While, for CD8+ cluster 1(CD8-C1), the expressions of tissue resident marker CD69, NR4A1, ZNF683 are very high, suggesting they are more likely to be tissue resident CD8+ T cells in Fig. 3d in the revised manuscript. And the tissue resident CD8+ T cells are a population of CD8+ T cells that retain in peripheral tissues without recirculating in the blood [PMID: 31255505]. It is rare for tissue resident CD8+ T cells emergence into the circulation [PMID: 33755718]. The tissue resident CD8+ T cells might be generated from putative precursors like naïve T cells or effector memory T cells in the lymph nodes, then after differentiation and activation, finally reside into the tumor tissues [PMID: 29777219; PMID: 33755718; PMID: 31255505]. The trafficking and retention properties of tissue resident CD8+ T cells in different sites is still not clear [PMID: 33755718], and needed to further investigate. For kidneys, tissue resident CD8+ T cells has been found abundant [PMID: 33535505; PMID: 32973321; PMID: 29093030]. Consistent with this, we also discovered CD8-C1 cells as tissue resident CD8+ T cells in our study are significantly enriched in ART in Fig. 3e in the revised manuscript. But the mechanisms related to the high abundance of tissue resident CD8+ T cells in human kidneys are still unknown [PMID: 32973321]. Moreover, tissue resident CD8+ T cells could also be accumulated in epithelial tumor regions [PMID: 31255505], including ccRCC [PMID: 33861994], with little known regulating pathways [PMID: 33755718]. These results suggest tissue resident CD8+ T cells plasticity for residing in normal and tumor tissues. We guess that tissue resident CD8+ T cells in TTs may come from blood vessels, or lymphatics near to veins, or even adjacent primary tumors. Further studies are needed to investigate the origin of tissue resident CD8+ T cells. Moreover, researchers have found the retention ability of tissue resident CD8+ T cells and recruitment ability of certain tissues are influenced by different factors. According to the published studies [PMID: 33755718], CD103 is particularly important for tissue resident CD8+ T cells retention in tumors. Antibody blockade of CD103 or genetic deficiency of CD103 results in reduced tumor-infiltrating T cells and accelerated tumor progression in mice [PMID: 28738020; PMID: 28018343; PMID: 23408053; PMID: 26420854]. And certain* other integrins, including CD44, CD49a and CD69 might also help tissue resident CD8+ T cells retention in tumors [PMID: 33755718]. Another molecule is TGF-β considered a central mediator of tissue resident CD8+ T cell accumulation in tumors as well as in normal tissues [PMID: 26921343; PMID: 24162775; PMID: 26682984; PMID: 22504644; PMID: 30742629]. Since there is more tissue resident CD8+ T cells in TTs compared with PTs, we examined the expression levels of CD103, CD44, CD49a and CD69 on CD8-C1 cluster cells, and TGF-β on malignant cells between PTs and TTs in our single cell RNA-seq data, and found that the expression levels of all of them except CD44a are increased in TTs (Response Fig. 2A). And the TGF-β expression level is positively correlated with the abundance of CD8-C1 cells in TTs in our cohort of patients with paired PT and TT bulk transcriptomes (Response Fig. 2B). Therefore, we think the tumor cells in TTs might have a stronger recruiting ability to tissue resident CD8+ T cells, and the tissue resident CD8+ T cells themselves may prefer to reside in tumor thrombus compared with PTs.

*Response Fig. 2 CD103, CD44, CD49a and CD69 and TGF-β expression associated with CD8-C1 retention. (A). Dot plot showing the expression levels of CD103, CD44, CD49a and CD69 in CD8-C1 cluster, and TGF-β in malignant cells between PTs and TTs. (B). Scatterplots representing the correlation between TGF-β expression and the CD8-C1 ratio in the PTs and TTs of the validation cohort. The Pearson coefficient (R) and associated p value are reported for each correlation.*

*For exhausted T cells, it is predictable that a higher exhausted CD8+ T cells are in PTs and TTs, which is consistent with one study published recently [PMID: 32396851]. Unfortunately, we do not find the difference about exhausted CD8+ T cells between PTs and TTs. But for CD4+ Tregs, we found the relative percentages of suppressive Tregs (CD4-C4) in PTs were higher than those in ARTs and TTs indicating that the immunosuppressive state might be different in PTs and TTs.*

6. There are no mulitregional profiling performed and previous effort from TracerRX cohort showed that the TT can be seeded in different mechanism (ie early event vs advance subclones). Can the authors utilize either their discovery or validation cohort to comment on ITH and the TME with both the PT and TT.

*Response: We appreciate your great comment. According to the reviewer’s comment, we try to assess the intratumoral heterogeneity (ITH) of PTs and TTs which may reflect the dynamics of cancer cell evolution. We use a method based on one published study [PMID: 31588021] to compute tumor cell specific transcriptomic diversity score for measuring the degree of intratumoral heterogeneity, via the gene expression profiles of malignant cells within the tumor. And we find that there are 4 patients with increased ITH in TTs compared with matched PTs, four patients with lower ITH than those in paired PTs, and one patient with little change of ITH between PT and TT (Fig. S2a in the revised manuscript). The different diversity changes of ITH between TTs and matched PTs suggests that unlike metastases harbor fewer somatic alterations than primary tumors [PMID: 29656895; PMID: 27124450], TTs are not always seeded from few advanced clones of PT.*

*Then, we construct the phylogenetic trees of each patient by Uphyloplot2 based on InferCNV results, and find that in most of patients, many subclones include both TTs and PTs malignant cells suggesting that these tumor cells in TTs come from PTs except P06 patient which are consistent with our results in the manuscript (Fig. 2a). Furthermore, we observed that various degrees of CNV events occurred in the subclones. For example, in Response Fig. 3, in P01, the clone L contains many different CNV events, while clone K obtains less CNV events. Two of them have similar proportions of malignant cells. But in P08, the clone D with amounts of tumor cells have less CNV event compared with clone E. While, in P06 the clone I with amounts of tumor cells have more CNV event compared with clone H. So, it is difficult to conclude that TT may be seeded from the subclone with early CNV events or advanced subclone. Moreover, one paper published recently showed that that TT formation is not associated with the most aggressive clone, but driven by immediate early genes, it might be an opportunistic trait attained by subclones with diverse oncogenomic status in geospatial proximity to vasculature [PMID: 34608135].*

*Response Fig. 3 Phylogenetic trees of the 9 patients. The branches are scaled according to percentage of cells in the calculated subclone containing the corresponding CNVs. In addition, we have added the results of ITH scores in fig. S2a. Moreover, we inserted the information about how to calculate the ITH scores in the method section (page 25, line 537-544), and modified the sentences in the main text in the revised manuscript (page 5-6, line 108-115).*

7. In CellPhoneDB analysis, how do the authors justify doing analysis between only major cell types given so many clusters existing within each cell type? And how can we interpret the results from pooling cells with different phenotype or functional state?

*Response: Thanks for your constructive comments. We agree with the reviewer that it is reasonable to identify the interaction between sub clusters of the interested cell types. According to the reviewer’s comment, we tried to use the ligand-receptor pairs in Fig. 6a in the manuscript to explore the interaction in the sub cluster of myofibroblasts, epithelial cells and endothelial cells, since there is a strong interaction in ECM related ligand-receptor pairs between myofibroblasts and epithelial cells, and myofibroblasts and endothelial cells. From Fig. S6a in the revised manuscript, we can find that all of the myofibroblast subclusters have stronger interactions with the subclusters of tumor cells and ECs in these ligand-receptor pairs of ECM remodeling pathway. Although the cells in each cell type is divided into several sub clusters with different function, ECM related pathways were activated within many subgroups of the different cell types in TT compared with in PT. Therefore, the interaction of ECM related ligand-receptor pairs is still stronger in the different cell types in TT. Furthermore, we have added the results of the interaction in the sub cluster of myofibroblasts, epithelial cells and endothelial cells in fig. S6a, and modified the sentences in the main text in the revised manuscript (page 17, line 365).*

*8.* Is it reasonable approach to use the top 50 DEG genes (page 25) for building the signature of a specific cell cluster/function state without any filtering? How do the authors propose to avoid signal from other cell types which were not included in the DEG analysis? For example, immune related signature genes which are seen with signal from epithelial cells?

*Response: We appreciate your great comment. According to the reviewer’s suggestion, we excluded genes with average expression < 2 in each cell type from each top 50 DEGs candidate marker set, and manually reviewed the expression patterns of these genes by dot plot and feature plot. And we removed those that were obviously expressed in many other cell types. Furthermore, we calculated the cell type specific score for tumor cells, endothelial cells and myofibroblasts which were used in Fig. 6c-6f in the manuscript with each screened cell marker set, and the score calculated by the relevant markers was the highest and obviously significant in the corresponding cell population (Response Fig. 4). In addition, we have updated the new figures in fig. 6c-6f and fig. S6b-S6d, and modified the sentences in the methods section in the revised manuscript (page 26, line 565-566).*

*Response Fig. 4 Box plots showing the signature score distribution for tumor cell, endothelial cell, and myofibroblast within the cell types that we identified in our single cell RNA-seq data. p values were determined by a two-sided Wilcoxon test.*

9. The parameter MAST (hurdle model) in the FindAllMarkers function was used to perform DEG analyses (page 24) which I believe is not taking into account of possible batch effect from patients and sample types. The author should consider consider using "negbinom" or "LR" parameter which allow incorporating the patient and the sampling site (ART, PT or TT) as a covariate in the DEG model?

*Response: We appreciate your great suggestion. During performing DEG analysis, according to some published single cell RNA-seq papers [PMID:32767996; PMID: 34489460; PMID:34489465; PMID:34083450; PMID: 34437124; PMID: 34162860; PMID: 32398875], we used the MAST algorithm provided within the FindAllMarkers function with default parameters [PMID: 26653891]. Furthermore, according to the reviewer’s suggestion, we performed differential gene expression analysis between different subgroups by using negbinom algorithm within malignant cells and myofibroblasts, and obtained consistent results with those in the manuscript (Fig. S2g and Fig. 5o). In Response Fig. 5A, antigen processing and presenting or IFN-γ response pathways are enriched in PTs compared to TTs, while, ECM related pathways and response to metal ion pathways are activated in TTs when we used the using "negbinom" parameter for DEG analysis. Furthermore, we found the overlap ratio between our DEGs and DEGs using “negbinom” parameter in malignant cells is about 76%. In addition to this, in Response Fig. 5B, most subclusters of myofibroblasts in TTs also increased the expression signatures associated with ECM remodeling, including EMT, extracellular matrix organization, and collagen fibril organization and EMT pathways, which is highly consistent with those in the manuscript. The overlap ratio between with or without “negbinom” parameter in myofibroblasts is 78%. So, based on these papers and results, we think it is feasible to perform DEG analysis with the parameter MAST (hurdle model) in the FindAllMarkers function.*

*Response Fig. 5 GSEA analysis of subclusters of malignant cells and myofibroblasts. (A). The IFN-γ signaling pathway or antigen presentation and processing (Antigen Proc. and Pres.) enrichment scores in clusters 1-4 of malignant cells in PTs compared with those in TTs; extracellular matrix remodeling-related pathways or response to metal ion enrichment scores in clusters 1-4 of malignant cells in TTs compared with those in PTs. (B). Heatmap of GSEA enrichment scores for indicating gene sets significantly enriched in PTs or TTs. EMT, epithelial- mesenchymal transition.*

**Second round of review**

**Reviewer 1**

Shi et al provide a resubmission of "Decoding the multicellular ecosystem of vena caval tumor thrombus in renal cell carcinoma by single-cell RNA sequencing." In this revision, the authors have address many of the minor points but have not addressed some of the broader issues at hand from both reviewers (several were not discussed in the response to the reviews letter). The strengths are the important resource that has been generated. However, there remain a number of inconsistencies and while the themes in the sections seem fine, the text supporting the themes needs organization. Finally, there are some very strong conclusions made from transcriptome data which would need further validation.

General comments
1) Still the discussion of the patient with TFE-RCC adds to the confusion - particularly in figure 1 and 2

2) The authors introduce concepts and ideas without enough setup for a non-kidney researcher. E.g. there are more than just proximal tubule cells and so there is a lack of discussion about the ARTs before going into the comparisons of tumor/thrombus/adjacent. What happened to the other 25k ART cells? Then in subsequent analyses, are the authors using the 2k ART cells or all ART cells?

3) Despite a lot of information presented, key results are not easily drawn out. The manuscript would benefit from simplifying concepts and improved organization. Some subsections read well while others are a long list of gene names or summary of background information that could be summarized to make it easier for a reader.

4) Conclusions need to be pulled out of the results and put in the discussion/conclusions (Eg lines 225-232)

5) Conclusions may be overinterpreting the data - eg 429-431 - without liquid biopsies, this conclusion is difficult to make. THe same with 450-451 - again, the conclusion that one could address a tumor thrombus with targeting CD248 is not proven here. Only that there is an enrichment in TTs.

**Reviewer 2**

Satisfied with response.

**Authors Response**

**Point-by-point responses to the reviewers’ comments:**

Point-to-point responses ------------------------------------------------------------------------------------------------------- -------------------- We thank the reviewer for the insightful comments and suggestions, which have significantly improved our manuscript. Below are the point-to-point responses (blue text) embedded in the reviewer’s comments. In the second revised version, we have removed the TFE3-RCC samples as suggested, and re-analyzed the data from ccRCC patients, including T/NK cells, myeloid cells, malignant cells, endothelial cells and myofibroblasts. In brief, the key findings observed in ccRCC tumor thrombus were consistent with the results of TTs in the previous version of manuscripts, suggesting that these findings in ccRCC tumor thrombus are indeed existing, with slight influence by the samples from TFE3-RCC patient. According to the reviewer’s comments, we also updated the description and the figures of cell types from ARTs, especially for the “normal epithelial cells” in ARTs. In addition, we have reorganized and modified the manuscript especially the parts for the CD8+ T cells, endothelial cells, myofibroblasts, and the discussion, and highlighted the changes in red. Taken together, our study dissects the intratumoral multicellular heterogeneity of tumor thrombus in clear cell renal cell carcinoma, which provides novel insights into tumor thrombus formation, and would therefore be helpful for the development of effective targeting therapy. ------------------------------------------------------------------------------------------------------- --------------------

Reviewer #1:

General comments

1) Still the discussion of the patient with TFE-RCC adds to the confusion - particularly in figure 1 and 2.

*Response: We thank the reviewer for the insightful comment. We do agree with the reviewer that the clinicopathologic, genomic and transcriptomic features of TFE3-RCC subtype are very different from ccRCC. Moreover, one research observed lower levels of immune infiltration signatures (IIS) in TFE3-RCC in contrast to KIRC of the TCGA cohort from bulk RNA-seq data [PMID: 34489456], suggesting the tumor immune microenvironment of TFE3-RCC patients is significantly different from that of ccRCC patients. However, unfortunately, owing to only one patient related to TFE3-RCC in our single cell RNA-seq data, we could not identify the statistically significant differences between TFE3-RCC and ccRCC at single cell resolution. Therefore, as the reviewer suggested, and considering the dramatic differences between TFE3-RCC and ccRCC, we removed the samples of the TFE3-RCC patient from our discovery cohort, re-analyzed the data of ccRCC samples, and replaced accordingly all the figures and tables in the main text and in the supplementary files in the second revised manuscript. In brief, the core findings in TTs of the ccRCC samples are in accordance with the results of our previous manuscripts, and impacts from TFE3-RCC samples are existing, Response letter Click here to access/download;Personal Cover;P2P-Reviewers comments for TT paper 20220118-2.docx but relatively small on our main conclusions. Here, the key findings in the second revised manuscript are listed as follows:*

1. *The CD8-C1 cluster, characterized by expression of tissue resident CD8+ T cell markers, was enriched in TTs compared with in PTs, which resembled a progenitor exhausted phenotype. High levels of CD8-C1 signature were significantly associated with improved PFS after ICB therapy.*
2. The macrophages as the tumor associated macrophages (TAMs) were enriched in PTs and TTs compared with ARTs. The TAMs in PTs showed a pro-inflammatory phenotype, but increased the expression of immunosuppressive markers compared with TTs, while the TAMs in TTs enhanced activities in tumor progression-related pathways including extracellular structure organization, EMT, angiogenesis and glycolysis.
3. Like the macrophages, the malignant cells from PTs exhibited both proinflammatory and anti- inflammatory phenotypes compared to TTs. And the tumor cells from TTs showed a higher ECM reprogramming state.
4. Almost all the subsets of endothelial cells and myofibroblasts in TTs showed increased activities of ECM remodeling related pathways relative to PTs. The higher proportion of Endo2 in TTs was associated with poor survival. And the Myo2 cluster signature enriched in PTs and TTs were associated with improved PFS with sunitinib therapy in the validation cohort.
5. For the cell-cell interactions, the ECM remodeling related interactions were stronger in TTs than that in ARTs and in PTs, and the tumor-stroma interplay was associated with poor survival.

Therefore, our previously submitted core findings have been consistently validated in this second revised version. Furthermore, we also found some small differences from removing the samples of the TFE3-RCC patient in the second revised manuscript:

1. After reclustering the CD8+ T cells, we obtained two exhausted CD8+ T cell subsets, CD8-C2 and CD8-C3. And the cells of CD8-C3 are enriched in both PTs and TTs than in ARTs, and not different from each other (Fig.2e). Meanwhile, there is no difference in the proportion of CD8-C2 subset across ARTs, PTs and TTs (Additional file 1: Fig.S2e). These findings in the second revised manuscript were consistent with those in the previous version of manuscripts.
2. After reclustering the myeloid cells, though the number of myeloid cells subclusters was changed, we observed almost all the subsets of macrophages in PTs exhibited increased scores of antigen presentation and interferon response pathways, but more activated in tumor progression related pathways in TTs, which was shown in Fig.3g in the second revised manuscript. And this updated figure provides stronger support for the different stats of TAMs between PT and TTs than that of the previous version. Moreover, these macrophages were still TAMs and enriched in PTs and TTs compared to ARTs (Fig.3d and Additional file 1: Fig.S3e). All these findings were in accordance with those of previous manuscripts.
3. The classic ferroptosis suppressor gene MT1G was not observed increased in the malignant cells of TTs after removing the data of TFE3-RCC. Furthermore, we have instead of the figure of this gene with MT1X (Additional file 1: Fig.S4h), and deleted the sentence related to MT1G in the result section of the second revised manuscript (page 11, line 233).
4. After reclustering the ECs, we identified six the ECs subclusters. Most of the subclusters of ECs in TTs exhibited enhanced activates of collagen fibril organization, extracellular structure organization and cellular matrix adhesion compared to PTs (Fig. 5g and Additional file 1: Fig.S5g), which further support our discovery that ECM remodeling is more enhanced in tumor thrombus. And, the proportion of ACKR1+ ECs (Endo4) had a downward trend, but not significantly decreased in PTs and TTs compared with ARTs (Additional file 1: Fig. S5d). The peritubular-related ECs were not identified in this version, due to they are the least in ECs (only 1.8% of the ECs), and are mainly enriched in adjacent renal tissues, after removing the ART (a quarter of the total ARTs), PT and TT samples of TFE3-RCC, they become less and are probably filtered out during analysis. But, the key findings about Endo2 subset in the previous manuscript are confirmed in this updated manuscript, that the cells of Endo2 subset (cancer-related ECs) were enriched in TTs and the high signature of them was associated with poor survival.

Furthermore, we have modified the description according to our new results and highlighted in red in the second revised manuscript. Taken together, there are some slight changes in our second revised manuscript, but the key findings in our previously submitted version have been consistently validated in this updated manuscript.

The authors introduce concepts and ideas without enough setup for a non-kidney researcher. E.g. there are more than just proximal tubule cells and so there is a lack of discussion about the ARTs before going into the comparisons of tumor/thrombus/adjacent. What happened to the other 25k ART cells? Then in subsequent analyses, are the authors using the 2k ART cells or all ART cells?

*Response: We are very sorry for the unclear description in the manuscript. We have followed this suggestion to update the figures including the marker genes expression of the identified major cell types and their compositions in ARTs, PTs and TTs (Fig. 1f-g), and further added the related description and the relevant reference at the introduction section of the second revised version (page 4-5, line 87-90). Moreover, UMAP plots were also included to represent the identified proximal tubule cells and the loop of Henle cells in ARTs with the previously described classic markers [PMID: 30093597] in this version (Additional file 1: Fig. S1d). The reason why we use proximal tubule cells for comparison with malignant cells is that they have been reported as the origin for ccRCC [PMID: 30093597]. Thus, we only used the proximal tubule cells from ARTs to compare with the malignant cells from PTs and TTs for calculating the hypoxia and angiogenesis signature scores, and observed significant increases in PTs and TTs (Fig. S4b). To avoid the proximal tubule cells disturbing our understanding of tumor cell heterogeneity across each subcluster, and to better understand the malignant cell differences between PTs and TTs, then, we only resubclustered the malignant cells, and investigated the four malignant cell subclusters by GSVA Hallmark analysis. Furthermore, in the last result subsection of the manuscript, we also compared the interactions of the proximal tubule cells with TAMs, ECs and myofibroblasts, and the interactions of malignant cells with TAMs, ECs and myofibroblasts on ECM remodeling related ligands and receptors, and observed their interaction in TTs was stronger than those in ARTs and PTs (Fig. 6a and Fig. S6a). Furthermore, according the reviewer’s comment, we added this detailed description and the relevant references in the main text (page 10, line 208-211) and in the legends of Fig6a (page 34, line 670-671), and updated the supplementary figure (Fig. S6a) in the second revised manuscript.*

Despite a lot of information presented, key results are not easily drawn out. The manuscript would benefit from simplifying concepts and improved organization. Some subsections read well while others are a long list of gene names or summary of background information that could be summarized to make it easier for a reader.

*Response: We appreciate this very helpful suggestion. We have followed this to reorganize the result subsections of the manuscript, in the order of T cells, macrophages, malignant cells, endothelial cells and myofibroblasts. In addition, we also simplified some statements in the sections of T cells, macrophages, malignant cells, and the discussion, and modified the figures and the statements of endothelial cells. Besides that, we have shortened the gene names of the T cells (page 5-6, line 109-111), endothelial cells (page 11, line 246-247) and myofibroblasts (page 12, line 275-278), and introduced each subcluster briefly. Furthermore, all the changes in the second revised manuscript were highlighted in red.*

Conclusions need to be pulled out of the results and put in the discussion/conclusions (Eg lines 225-232)

*Response: Thanks for this helpful suggestion. These statements been simplified to one sentence in the result section of the second revised manuscript (page7, line 153-155). Furthermore, we have moved other statements discussing tissue resident CD8+ T cells to the discussion section. (page 18-19, line 390-400)*

Conclusions may be overinterpreting the data - eg 429-431 - without liquid biopsies, this conclusion is difficult to make. THe same with 450-451 - again, the conclusion that one could address a tumor thrombus with targeting CD248 is not proven here. Only that there is an enrichment in TTs.

*Response: Thanks for pointing out this. We have followed this suggestion to modify the statements in the discussion section of the second revised manuscript (page 17, line 366-368) as the following: “These results suggest that malignant cells of TTs are not independently suspended in the blood vessels, but require to form an ECM network embedded with immune cells and stroma cells to promote tumor thrombus growth.” Additionally, to avoid overinterpreting the results about CD248, and to focus more on the key findings about enhanced ECM remodeling and increased tissue resident CD8+ T cells in TTs, we removed some content and modified the statements of our discussion of the second revised manuscript. Furthermore, all the changes are highlighted in red in the second revised manuscript.*

The list of changes for Decoding the multicellular ecosystem of vena caval tumor thrombus in clear cell renal cell carcinoma by single-cell RNA sequencing

1. We have replaced the title of “renal cell carcinoma” to “clear cell renal cell carcinoma”, after removing the TFE3-RCC samples in the second revised manuscript. (page 1, line 1-2)

2. The abstract has also been modified accordingly, including the sample size, and description of the key findings related to increased tissue resident CD8+ T cells and enhanced ECM remodeling in TTs, as well as the proinflammatory and immunosuppressive states of PTs. (page 1-2, line 19-26; page 2, 28)

3. In the keywords, We instead of “neoadjuvant therapy” and “Kidney cancer” with “Tumor microenvironment” and “Clear cell renal cell carcinoma”. (page 2, line 32- 33)

4. The sentence of “accounting for approximately 70–80% of all RCC cases” and the relevant references are added in the background section. (page 2, line 38)

5. The statements for the background information based on genomic and bulk RNAseq analysis are also be simplified. (page 3, line 50-51, 53-56, 58-59, 62-63)

6. The summary statements of our study are also updated and modified. (page 3, line 64; page 4, 69, 70-71)

7. The introduction statements in the beginning of the result section are also updated and modified. (page 4, line 74, 76, 79-81, 85-86)

8. The description about cell composition of ART samples was also added. (page 4-5, line 87-90)

9. As the reviewer’s suggested, we reorganized the result subsections of the manuscript. In detail, they are in the order of T cells, macrophages, malignant cells, endothelial cells and myofibroblasts.

10. The description of T/NK cells results in the result section is simplified and modified. (page 5, line 93-97, 98-99, 102, 108)

11. The gene names of identified CD8+ T cell subsets are shortened. (page 5-6, line 109-111)

12. The description of CD8+ T cell subsetsresults in the result section is modified. (page 6, line 114, 119, 121, 123, 125-129)

13. The summarized statements about CD8+ T cells in PTs and TTs are added in page 6, line 129-131).

14. The description about the progenitor exhausted CD8+ T cells is simplified in page 6-7, line 132-136; page 7, line 148, 151-155).

15. The description of macrophages results in the result section is simplified and modified. (page 8, line 156-162, 164, 166, 174; page 9, line 180-181, 183, 186-189, 194-196)

16. The description of malignant cells results in the result section is simplified and modified. (page 9-10, line 197-202; page 10, 203, 207-211, 213, 217-218; page 11, line 223, 226-228, 233-237)

17. The description of ECs results in the result section is simplified and modified. (page11, line 238-240; page 11-12, line 242-244; page 12, line 245-248; page 12, line 251, 253, 255-265)

18. The description of myofibroblasts theme in the result section is simplified and modified. (page 13, line 267-268, 273, 275-278; page 13-14, 286-288; page 14, 289- 291, 297-299)

19. The statements of cell-to-cell interactions results in the result section are modified and simplified. (page 14, 308-309; page 15, line 315-316; page 15-16 329-334; page 16, 339-341, 343-344)

20. The statements of the discussion in the discussion section are modified and simplified. (page 16-17, line 350-354; page 17, 360-368; page 18, 376-382, 384- 386, 388-389, 390-395)

21. To avoid misleading the issue of batch effects, the detailed parameters, procedures and the relevant references are added in the method section. (page 20, line 426-438.

**Third round of review**

**Reviewer 1**

Overall, the rewriting and reorganizing of the data (after subtracting out the TFE-RCC case) has improved this manuscript significantly.

Minor comment is to carefully double check all the labels / files and supplementary data with the main text given so many moving parts. Otherwise, review text for colloquial language (e.g. line 155) and consider rewording.
